# Infectious Complications in Adults Receiving Extracorporeal Membrane Oxygenation: A Systematic Review and Meta-Analysis of Nosocomial and Bloodstream Infection, Prevalence, Risk Factors, and Clinical Outcomes

**DOI:** 10.3390/medsci14060653

**Published:** 2026-10-09

**Authors:** Aseel Alzibdeh, Ans Alamami, Sabeen Zara, Maha Aljabri, Rabee Tawel, Samar Hashim, Abdurrahmaan Elbuzidi, Hamad Abdelhadi, Ahmed Labib Shehatta, Nadir Kharma, Abdulsalam Saif Ibrahim, Ibrahim Fawzy Hassan, Muna Almaslamani

**Affiliations:** 1 Department of Infectious Diseases, Hamad Medical Corporation, Doha P.O. Box 3050, Qatar; aalzibdeh@hamad.qa (A.A.); szara1@hamad.qa (S.Z.); maljabri2@hamad.qa (M.A.); habdelhadi@hamad.qa (H.A.); malmaslamani@hamad.qa (M.A.); 2 Department of Medical Intensive Care, Hamad Medical Corporation, Doha P.O. Box 3050, Qatar; rtawel@hamad.qa (R.T.); aelbuzidi@hamad.qa (A.E.); ashehatta@hamad.qa (A.L.S.); nkharma@hamad.qa (N.K.); aibrahim1@hamad.qa (A.S.I.); ihassan@hamad.qa (I.F.H.); 3 Medical College, Qatar University, Doha P.O. Box 2713, Qatar

**Keywords:** extracorporeal membrane oxygenation, bloodstream infection, cross-infection, meta-analysis, immortal time bias

## Abstract

Background/Objectives: Reported infection rates during extracorporeal membrane oxygenation (ECMO) vary widely, partly because case definitions are applied inconsistently. We synthesized the prevalence and prognostic association of nosocomial and bloodstream infection (BSI) acquired during adult ECMO. Methods: PubMed/MEDLINE, Embase, Scopus and CENTRAL were searched through December 2025 for observational studies of at least 10 adults on ECMO with an explicit infection definition. We classified case definitions by the criteria actually applied. We pooled crude mortality odds ratios (ORs) and patient-level BSI prevalence in studies applying laboratory-confirmed criteria, assessed risk of bias with ROBINS-I and the JBI checklist, and rated certainty with GRADE. Results: Forty-seven reports (46 cohorts; 11,573 patients) were included. Pooled BSI prevalence was 25.8% (95% CI 17.3–36.5%; 7 studies, 1193 patients), 18.2% in pre-pandemic and 39.2% in COVID-19 cohorts (post hoc). Infection was associated with higher crude odds of death (OR 1.65, 1.33–2.04; prediction interval 0.95–2.85; 24 studies), but analyses modeling infection as time-varying were inconsistent, including no association in a multistate model (adjusted hazard ratio 1.00, 0.79–1.26), and small-study effects were suggested. Certainty was very low for both outcomes. Conclusions: Roughly one in four adults on ECMO developed laboratory-confirmed BSI, more often in COVID-19 cohorts. Whether infection acquired on ECMO increases mortality remains uncertain.

## 1. Introduction

Extracorporeal membrane oxygenation (ECMO) supports patients with refractory cardiac or respiratory failure through a circuit connected to large-bore intravascular cannulas, a blood pump and a membrane lung [1]. Veno-arterial (VA) ECMO provides circulatory support; veno-venous (VV) ECMO supports gas exchange. In adults with severe acute respiratory distress syndrome (ARDS), the CESAR trial showed that referral to an ECMO-capable center improved survival without severe disability [2], and EOLIA, although it did not meet its primary end point, favored early ECMO for very severe disease [3]; ECMO is now part of guideline-based ARDS care [4]. Use expanded during the H1N1 and COVID-19 pandemics, and by 2022 the Extracorporeal Life Support Organization (ELSO) registry had recorded more than 100,000 patients discharged alive [5].

Few critical care interventions concentrate on many infectious risks. The cannulas breach the skin, remain in place for days to weeks and cannot be exchanged routinely, because few vessels accommodate them and replacement carries procedural risk [1,6]. Contact between blood and the synthetic circuit activates complement, alters leukocyte phenotype and drives acquired immune dysregulation in patients who are already critically ill [7]. Prolonged mechanical ventilation, continuous renal replacement therapy (CRRT), high-volume transfusion, corticosteroids and other immunosuppressive agents, and long intensive care unit (ICU) stays add to the exposure [8,9,10,11]. Duration of support is the most consistently reported modifiable risk factor: in the ELSO registry, infection prevalence rose from 6.1% in runs of 7 days or less to 30.3% in runs longer than 14 days [12].

Reported infection rates during ECMO range from 8% to 64%, depending on population, configuration, case definition and surveillance method [6,8,9,10,11]. Registry analyses estimate 15 to 30 infections per 1000 ECMO-days, with adults carrying the highest burden [12]. Bloodstream infection (BSI) and ventilator-associated pneumonia (VAP) account for most nosocomial episodes [8,9,10,11].

Much of this eightfold spread is definitional rather than biological. The ELSO registry relies on center-reported positive cultures without standardized case definitions [13]. Agreement between experienced clinicians classifying infections during ECMO is poor [14]. A review of reporting practice proposed six minimum reporting elements that have not yet been widely adopted [15]. Studies that cite the same definition often apply it differently, and synthesizing that pool across definitions inherit the heterogeneity. The uncertainty carries over into treatment: ECMO alters antimicrobial exposure, and stewardship in this population still relies largely on evidence extrapolated from general intensive care [16].

Attributing mortality to infection raises a structural problem. Most cohorts classify patients as ever or never-infected. A patient must survive on support long enough to acquire an infection and have it ascertained, so this design favors higher mortality in the infected group unless infection is modeled as a time-dependent exposure [6]. The primary literature reflects this tension. Schmidt and colleagues reported nearly twofold higher adjusted odds of death among 220 adults on VA-ECMO [10], Aubron and colleagues found longer ICU and hospital stays without a significant mortality difference [9], and Sun and colleagues described a mixed signal in an early adult cohort [11].

We therefore conducted a systematic review with targeted meta-analysis of nosocomial and bloodstream infections acquired during adult ECMO. The review departs from earlier work in three ways: case definitions were classified by the criteria actually applied rather than by the definition cited; pooled prevalence was restricted to a definitionally harmonized subset; and the mortality association was interpreted in the light of immortal-time bias.

## 2. Materials and Methods

### 2.1. Protocol Registration and Reporting

The review was registered with PROSPERO (CRD420261442366) [17] and is reported in accordance with the PRISMA 2020 statement (Supplementary Table S1). The population was adults supported by VA- or VV-ECMO. The exposure was nosocomial infection or BSI acquired during ECMO support; the comparator, where reported, was ECMO-supported patients without such infection. Outcomes were infection prevalence and incidence, pathogen distribution, risk factors for infection, in-hospital and ICU mortality, ICU and hospital length of stay, ECMO duration, and weaning or decannulation success.

The protocol was registered on 6 July 2026, after the search and study selection were completed, and the review is therefore not prospectively registered. The record was amended after registration (versions 2.0 and 3.0, both published on 1 October 2026) to report the final study counts and statistics and to record the following deviations, none of which is presented as a prespecified method. (i) Information sources: Scopus, which was searched together with the other databases, was added to the record; ClinicalTrials.gov, listed at registration, was not retained as an information source, and no record from it enters the flow diagram; the searches combined the ECMO and infection concepts only, without the outcome block described in the protocol, which can only increase sensitivity. (ii) Age: five cohorts that included, or may have included, patients younger than 18 years were retained as adult-predominant (Section 2.2). (iii) Eligibility: two conditions identified during source verification were added, namely ECMO as the entry criterion and separability of the ECMO population, applied per outcome (Section 2.2). (iv) Re-audit of full-text exclusions: re-examination of every full-text exclusion against the eligibility criteria reinstated six reports that had been excluded in error (Section 2.4). (v) Case definitions: the operational Tier A–C framework was introduced before any pooled estimate was computed (Section 2.7). (vi) Outcome A: the rule of including every study with reconstructable 2 × 2 mortality data was re-applied to all reports in successive audits, which increased the primary set from 11 to 24 studies; post hoc sensitivity, influence and subgroup analyses were added, including a pooled comparison of COVID-19 with pre-pandemic cohorts for both outcomes; only a descriptive COVID-19-era comparison had been prespecified (Section 2.9). (vii) Pooling models: the registered DerSimonian–Laird model with Mantel–Haenszel estimation (Outcome A) and the Freeman–Tukey double-arcsine transformation with DerSimonian–Laird estimation (Outcome B) were replaced by REML with Hartung–Knapp intervals and a logistic-normal GLMM, respectively; estimates from the registered methods are reported alongside (Section 3.9 and Section 3.10); Peters’ test, planned for Outcome B, was not performed because it has little power with seven studies. (viii) Risk of bias: ROBINS-I was applied, as registered, to all comparative results; the JBI prevalence checklist was added for the prevalence evidence and applied to all reports, and the Cochrane RoB 1 tool listed in the record was not applicable because no randomized trials were included (Section 2.8). (ix) Certainty: GRADE started at high rather than low certainty, in line with current guidance for evidence assessed with ROBINS-I and for event rates (Section 2.8). (x) Extraction: ECMO indication and antimicrobial prophylaxis protocol were added as variables at revision (Section 2.6). The registration record and its amended versions are available from PROSPERO (https://www.crd.york.ac.uk/PROSPERO/view/CRD420261442366, accessed on 1 October 2026).

### 2.2. Eligibility Criteria

Studies were eligible if they met all of the following: observational design (prospective or retrospective cohort, case–control or registry analysis); at least 10 adult patients receiving ECMO; an explicit case definition of infection (the definition applied by each included report is listed in Supplementary Table S2); at least one outcome of interest; and publication in English between January 2000 and December 2025. We excluded pediatric-only cohorts, case reports, conference abstracts, editorials, narrative reviews and reports without infection-specific outcomes.

We applied two additional conditions during source verification. ECMO had to be the entry criterion rather than a covariate within a broader critically ill population. The ECMO population also had to be separable from co-enrolled patients supported by other extracorporeal or mechanical circulatory devices; this condition was applied per outcome, so that a study contributed a given outcome only where that outcome was reported for the ECMO population alone. Five cohorts included, or may have included, patients younger than 18 years: three had a lower age bound of 16 years (Sun 2010 [11], Aubron 2013 [9] and Wang 2023 [18]), one reported an age range of 16.7 to 77.7 years (Haneke 2016 [19]) and one reported a lowest age quartile of 2 to 35 years (Grasselli 2017 [8]). None reported the number of patients younger than 18 years, and mean or median ages were 46 to 57 years. These cohorts were retained as adult-predominant, a deviation from the registration criterion whose influence was examined by sensitivity analysis.

### 2.3. Information Sources and Search Strategy

We searched PubMed/MEDLINE, Embase, Scopus and the Cochrane Central Register of Controlled Trials from inception to December 2025, with no date limits in the search strings; the January 2000 publication limit was applied at screening. Search terms combined ECMO concepts with nosocomial and bloodstream infection concepts, using controlled vocabulary where available together with free-text terms; the full strategy for each database, with the date it was run and the number of records retrieved, is given in Supplementary Table S3. The searches returned 6896 records (PubMed/MEDLINE 1543; Scopus 2981; Embase 2157; Cochrane CENTRAL 215).

### 2.4. Study Selection

**Figure 1:**
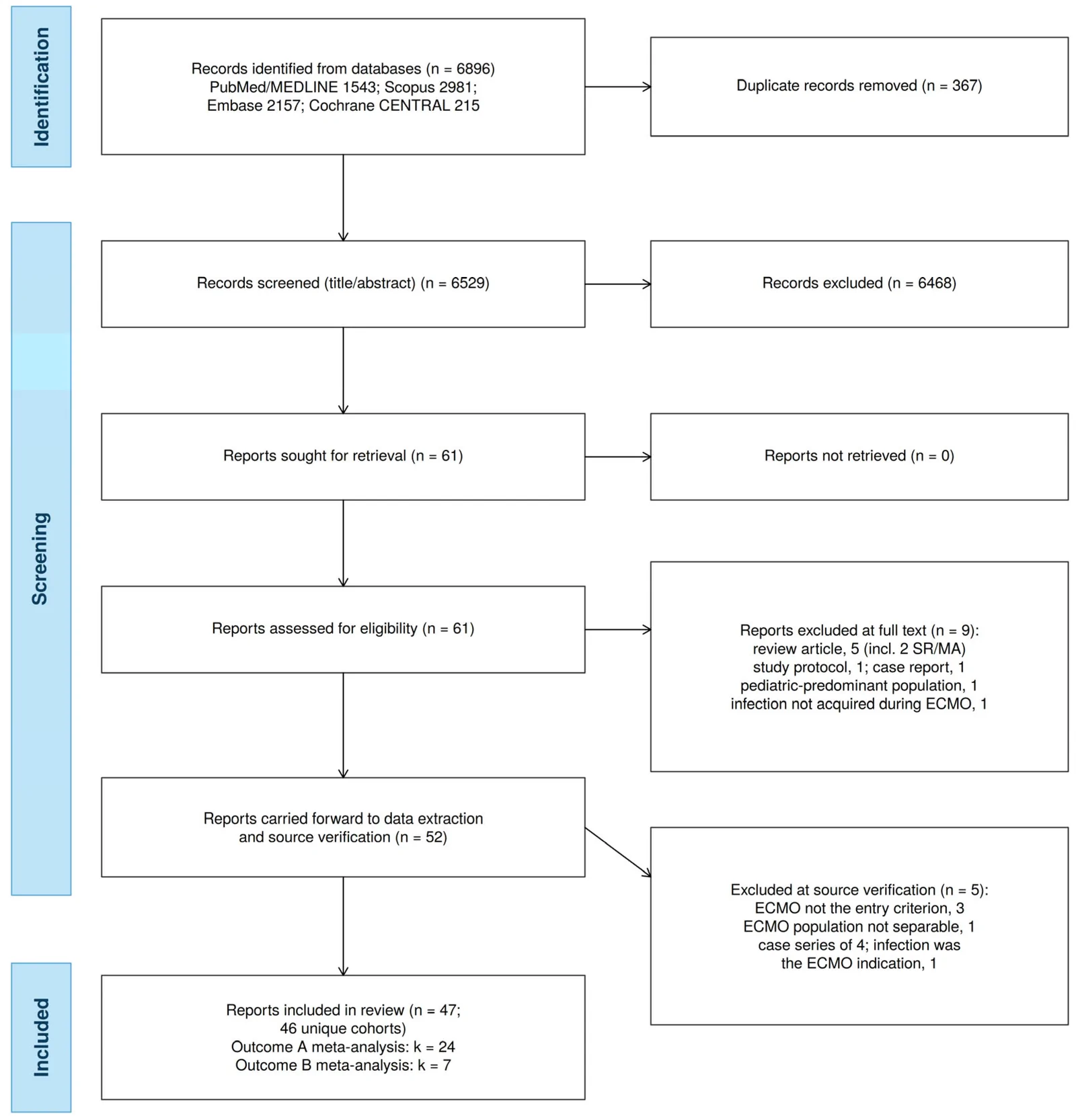
PRISMA 2020 flow diagram of study identification, screening, eligibility assessment and inclusion. Full-text exclusions are shown in two stages: the eligibility stage (9 reports) and the source-verification stage (5 reports). The 47 included reports describe 46 unique cohorts.

Two reviewers independently screened titles and abstracts, and a third arbitrated discordant judgments. After removal of 367 duplicate records, 6529 records underwent title and abstract screening, of which 6468 were excluded. Sixty-one full-text reports were assessed in two stages. At the full-text eligibility stage, we excluded nine studies: five review articles, one study protocol without results, one case report, one pediatric-predominant cohort without separable adult data and one report in which infection was ascertained only after decannulation. The remaining 52 reports underwent data extraction and source verification against the full text, at which stage five further reports were excluded: three because ECMO was not the entry criterion or the infection was not acquired during ECMO support, one because the ECMO population could not be separated from patients supported by other extracorporeal devices, and one because infection was the indication for ECMO rather than a complication of it. Six of the 52 reports had been excluded at the full-text stage of the original screen and were reinstated when every full-text exclusion was re-examined during revision (Section 2.1). Forty-seven reports were therefore included [8,9,10,11,18,19,20,21,22,23,24,25,26,27,28,29,30,31,32,33,34,35,36,37,38,39,40,41,42,43,44,45,46,47,48,49,50,51,52,53,54,55,56,57,58,59,60]. Two of them describe the same cohort [41,42] and were treated as one study, so the 47 reports describe 46 unique cohorts. Supplementary Table S4 lists the 14 excluded full-text reports with reasons, and Figure 1 shows the flow of records.

### 2.5. Unit of Analysis

The analytic denominator was not uniform. Forty-three reports analyzed patients and four analyzed ECMO runs or procedures. Patient-level and run-level denominators are not arithmetically commensurable and were not summed; the included reports contributed 11,573 patients and 2220 ECMO runs or procedures, which are reported separately. In the patient total, the overlapping Pieri cohort is counted once; the reports from Beijing Anzhen Hospital [36,55] have overlapping enrollment windows that could not be reconciled at the patient level, so the total may include up to 322 patients twice. Two reports from mixed-age cohorts contributed their adult subgroup only 60 of 126 patients [48], another study included 31 of 92 [49]. Where a study reported infection episodes, isolates or positive cultures rather than infected patients, the numerator was recorded as episode-based and excluded from patient-level prevalence; four reports were affected [28,36,55,57].

### 2.6. Data Extraction

Data were abstracted into a piloted form capturing 27 variables per study: design, ECMO mode, demographics, case definition and ascertainment window, microbiology, site-level infection distribution, ECMO duration, length of stay, mortality and its endpoint, weaning success, reported risk factors, and predictors of adverse outcome. We resolved discrepancies by consensus, with adjudication by a third reviewer. During revision, we added ECMO indication and antimicrobial prophylaxis protocol as extraction variables (Section 2.1), and reaudited every value used in a table or pooled analysis against the primary publication. Corrections from that audit are recorded in the data-lock workbook, which the corresponding author can provide on request.

### 2.7. Classification of Infection Case Definitions

We classified each definition by the criteria actually applied rather than by the definition cited, because verification showed that the two often diverge. Of seven definitions first audited in detail, five were reclassified: three studies citing “CDC/NHSN” had applied the 1988 CDC definitions [61], which predate the NHSN laboratory-confirmed bloodstream infection (LCBI) framework; one cited CDC/NHSN but relied on metagenomic sequencing without a culture criterion; and one cited “CDC” but defined nosocomial infection only as infection arising more than 24 h after admission, a timing rule rather than a case definition.

Three tiers were defined after protocol registration and before computing any pooled estimate; the framework is therefore declared as a post hoc operational classification (Section 2.1) and was applied to all 47 reports. Tier A denotes LCBI logic: a recognized pathogen in at least one blood culture, with common skin commensals requiring at least two cultures drawn on separate occasions together with compatible clinical signs. Tier B denotes culture-based ascertainment without this commensal rule; such definitions can admit single-culture commensals to the numerator and may therefore over-count BSI. Tier C denotes non-culture ascertainment. Studies that quantified a different construct, or did not quantify BSI, were not assigned a tier. During revision, we reaudited every Tier A and Tier B assignment against the source text; one study [11] was reclassified from Tier A to Tier B because it counted a single commensal culture as BSI when clinical signs were present. The operational criterion, its source and the resulting tier for each report are listed in Supplementary Table S2.

### 2.8. Risk of Bias Assessment

We used two tools. The Risk of Bias In Non-randomized Studies of Interventions (ROBINS-I) tool [62] was applied at the level of the result to every report that compared mortality (or, in one report, ECMO weaning) between infected and uninfected patients: 37 results, comprising the 24 in the primary mortality meta-analysis, 2 entering sensitivity analyses only and 11 reported narratively; the adjusted time-dependent estimates of two reports were assessed as additional narrative results. It was not applicable to the 10 reports without such a comparison. Because ROBINS-I assesses bias in an effect estimate rather than a descriptive proportion, the Joanna Briggs Institute (JBI) critical appraisal checklist for prevalence studies [63] was applied to all 47 reports. For item 3 (adequate sample size), the threshold was 246 patients, the number needed to estimate a prevalence of 20% within ±5% with 95% confidence. For item 7, cultures obtained on clinical suspicion rather than by systematic surveillance were rated unclear. Reports were classed as low concern when at least seven of nine items were met, moderate when five or six were met, and high when four or fewer were met, and a report was moved one category toward greater concern when the condition was not measured in a valid way for all participants (item 6). One reviewer made preliminary ratings, while the second verified them; disagreements were resolved through discussion. Item-level JBI judgments and domain-level ROBINS-I judgments are presented with the results section.

Certainty of evidence was rated with GRADE [64]. In line with current guidance, evidence assessed with ROBINS-I started at high certainty and was downgraded for risk of bias, as the tool indicated [65]; the prevalence estimate, an event rate from observational cohorts, also started at high certainty [66]. The registered protocol had specified a low starting point for observational evidence; this change is declared in Section 2.1 and does not alter either final rating.

### 2.9. Data Synthesis

The evidence base shows substantial clinical, methodological and definitional heterogeneity, so synthesis was delivered in two layers: a narrative synthesis with descriptive statistics covering all 47 reports, and a targeted meta-analysis restricted to two outcomes for which pooling was defensible, the mortality odds ratio (OR; Outcome A) and patient-level BSI prevalence within the definitionally harmonized subset (Outcome B).

Seven reports contribute to the narrative synthesis but not to the descriptive summary statistics, because each fails a denominator, comparator or population-separability condition [22,25,40,42,43,56,60]; the remaining 40 contribute to those statistics. We report continuous study-level variables as the median with interquartile range (IQR) and range across studies. These values describe the distribution of published study-level estimates, are not weighted for sample size and are not pooled estimates. A study contributed a value only when it reported a single cohort-level value or one that was arithmetically derivable. Every data cell in Table 1 carries one of three values: a numeric value, as reported or derived by the review team; NR, not reported in the source, including values available only for a subgroup other than the one extracted; or NA, not applicable, where the item cannot exist for that report by design. We converted durations reported in hours to days. We harmonized mortality to the in-hospital endpoint, treating survival to discharge as its complement, and accepted mortality without a stated time frame as reported; we excluded reports that provided only ICU mortality, death on ECMO, one-year mortality, or mortality restricted to infected patients from that summary. We classified each study as VV-predominant (VV only or VV at least 70%), VA-predominant (VA only or VA at least 70%), or mixed. Because microbiology was reported at varying granularity, we could not calculate organism-specific and site-specific prevalence; we report these qualitatively.

For Outcome A, every study from which infected-group and non-infected-group mortality could be reconstructed as 2 × 2 counts was eligible, irrespective of exposure construct or unit of analysis; 24 qualified. No study had a zero cell, so no continuity correction was applied. Log ORs were pooled with a random-effects model, estimating between-study variance by restricted maximum likelihood (REML) and computing confidence intervals (CIs) with the Hartung–Knapp method, which maintains nominal coverage better than Wald-type intervals when studies are few. The 95% prediction interval was based on a t distribution with k − 2 degrees of freedom. Two sensitivity analyses followed the registered outcome definitions: restriction of the exposure to BSI or bacteremia, and restriction of the end point to in-hospital mortality. The following were added post hoc: exclusion of studies analyzed by run or procedure; exclusion of cohorts that included, or may have included, patients younger than 18 years; exclusion of the single study whose exposure was invasive fungal disease; exclusion of the study with an outlying estimate from a sample selected on the availability of catheter-tip cultures [31]; exclusion of the influential study [39]; exclusion of the two studies whose 2 × 2 table was reconstructed from percentages or was internally inconsistent [10,48] and the alternative reconstruction for Schmidt 2012; addition of the report that gave a crude OR without cell counts [43] and of the report whose comparator was a propensity-matched sample [44]; leave-one-out and influence diagnostics; and exploratory subgroup analyses by exposure construct, by ECMO indication (respiratory including COVID-19, cardiac, or mixed) and by COVID-19 cohort status (at least 50% of patients with COVID-19). Small-study effects were examined with Egger’s regression, Begg’s rank correlation and Peters’ test, and with trim-and-fill using the L0 and R0 estimators. Pooled ORs from the registered DerSimonian–Laird and Mantel–Haenszel methods, and from Paule–Mandel and binomial–normal models, are reported for comparison.

For Outcome B, a study was eligible only if it met three conditions: a Tier A case definition, a patient-level BSI numerator, and a matching cohort denominator; seven qualified. Prevalences were pooled with a logistic-normal random-effects generalized linear mixed model (GLMM) fitted by maximum likelihood, which retains the binomial likelihood of each study and avoids the back-transformation problems of variance-stabilizing transformations. Heterogeneity is expressed as the standard deviation (τ) of the random effect on the logit scale, with a profile-likelihood CI to check the Wald interval. We report the registered Freeman–Tukey double-arcsine model with DerSimonian–Laird estimation for comparison. Sensitivity analyses excluded Pieri 2012 [41], whose sampling was case–control and whose BSI numerator could not be confirmed as wholly ECMO-acquired; substituted its later report (Pieri 2013 [42]); and added Sun 2010 [11]. We performed a post hoc comparison of COVID-19 with pre-pandemic cohorts as a subgroup analysis and as a GLMM meta-regression. We did not conduct small-study tests or mode-specific subgroup analyses because, with seven studies, these procedures have little power and unstable type I error.

Heterogeneity was quantified with Cochran’s Q (evaluated at *p* < 0.10), I^2^ computed from Q as (Q − df)/Q, and τ^2^. Where restricting a pooled set reduced τ^2^ to zero, the reduction is interpreted in the light of which studies were removed rather than as evidence of homogeneity. Overall nosocomial infection prevalence across mixed case definitions, mode-stratified BSI prevalence, between-group differences in ECMO duration and length of stay, and pathogen- and site-level prevalence were judged unsuitable for pooling and are reported descriptively.

### 2.10. Statistical Software

Analyses were run in R version 4.3.3 [67] with the metafor package (version 4.4-0) [68] and, for the GLMM, lme4 (version 1.1-35.1). The analysis script first reproduced the estimates of the previous version of the review and then added the reinstated reports. Two-sided *p*-values below 0.05 were considered statistically significant.

## 3. Results

### 3.1. Study Selection and Characteristics

Forty-seven reports describing 46 unique cohorts, published between 2009 and 2025, met the eligibility criteria (Table 1). Seven contribute to the narrative synthesis but not to the descriptive summary statistics [22,25,40,42,43,56,60]; the other 40 contribute to those statistics.

Retrospective cohorts predominated (38/47, 80.9%), including one retrospective analysis of prospectively collected data [8], followed by registry analyses of the ELSO database (4/47, 8.5%) [24,26,43,50]; three studies (6.4%) were prospective [23,39,52], one was ambidirectional [28] and one was a case–control study [41]. Multivariable analysis was performed in 38/47 (80.9%), most often using logistic regression or Cox proportional-hazards modeling. Four reports modeled infection as a time-varying exposure in analyses of death: Li et al. with a time-dependent Cox model [36], Nesseler et al. with a multistate model [39], and Grasselli et al. [8] and Schmidt et al. [10] with a time-dependent covariate in Cox models of death during ECMO and in the ICU, respectively. Cohort size spanned three orders of magnitude (median 105; IQR 61–284; range 15–2129). Forty-three reports analyzed patients. Four used ECMO runs or procedures: Aubron et al. (146 procedures in 139 patients) [9], Juthani et al. (100 runs, with each change in ECMO mode counted as a separate run) [29], Suzuki et al. (1871 adult runs) [50] and Thomas et al. (103 runs in 99 patients) [52]. Pluim et al. analyzed only the first run of each of 2067 adults and was therefore treated as patient-level [43].

Twenty-two reports enrolled a mixed indication population. Among the remainder, the predominant indication was respiratory failure or ARDS in eight, COVID-19 ARDS in six, cardiogenic shock in five, post-cardiotomy failure in four, extracorporeal cardiopulmonary resuscitation (ECPR) in one and trauma in one (Table 1).

**Table 1:** Characteristics of the 47 included reports (46 unique cohorts): design, study period, ECMO configuration, indication, cohort size, demographics, case-definition tier, BSI proportion, ECMO duration, mortality with its end point, antimicrobial prophylaxis and role in the pooled analyses, with a detail row for each report.

Study	Country	Design	Period	ECMO Mode	Indication	N	Age (y)	Male (%)	BSI Tier	BSI (%)	ECMO Duration (d)	Mortality, % (Endpoint)	Prophylaxis AB/AF	Pooled Role
**Andersen 2023 [20]**	Denmark	Retro cohort	2020–2021	VV 100%	COVID-19 ARDS	50	53	66	B	38.0	14.5 (med)	58 (IH)	R/R	A
**Case definition:** Bacteremia = growth of bacteria in blood culture + fever and/or raised CRP and/or raised PCT. No CoNS contaminant adjudication (CoNS 11/50 = 22% of the cohort). Not CDC/NHSN. [Institutional/culture-based]|**Infection sites:** BSI (38%), VAP (42%), CAPA (12%), CMV (20%), HSV (14%), *Candida* (12%)|**Pathogens:** CoNS, *P. aeruginosa*, *A. baumannii*, *E. faecium*, *Candida* spp., CMV, HSV, *Aspergillus*|**Resistance phenotype:** No (1 MRSA, respiratory isolate only; no MDR in blood)|**Polymicrobial:** Yes (36%)|**Risk factors for infection:** Longer ECMO duration, immunomodulatory therapy (e.g., tocilizumab) (multivariable: yes)|**Prophylaxis:** antibacterial—Piperacillin/tazobactam + vancomycin during VV-ECMO; antifungal—Fluconazole|https://doi.org/10.1111/aas.14228														
**Andreev 2023 [21]**	Russia	Retro cohort	2020–2021	VV 87%, VA 2%, other 11%	COVID-19 ARDS	125	48.7	72.8	B	NR	18.5 (mean)	90.4 † (IH)	NR/NR	Narrative
**Case definition:** Blood cultures drawn only when bacteremia/fungemia clinically suspected. No formal case definition; no contaminant adjudication (CoNS counted). [Institutional/culture-based]|**Infection sites:** BSI only (700 cultures, 250 positive)|**Pathogens:** CoNS, *K. pneumoniae* (XDR/PDR), *A. baumannii*, VRE, *Candida* spp., *Burkholderia*, others|**Resistance phenotype:** Yes—XDR/PDR *K. pneumoniae* and *A. baumannii*; vancomycin-R *E. faecium*; oxacillin-R CoNS|**Polymicrobial:** Yes|**Risk factors for infection:** ECMO duration, immunosuppression, RRT, transfusions, lymphopenia (multivariable: no)|**Prophylaxis:** antibacterial—No protocol described; antifungal—NR|https://doi.org/10.15360/1813-9779-2023-2-2265														
**Aubron 2013 [9]**	Australia	Retro cohort	2005–2011	VA 66%, VV 34%	Mixed (cardiac)	146 ^u^	46 ^a^	73.3	A	14.4	8 (med)	34.2 † (IH)	S/N	A
**Case definition:** CDC/NHSN modified; >48 h after ECMO initiation and ≤72 h post-decannulation [CDC/NHSN]|**Infection sites:** BSI (24), VAP (23), CAUTI (5)|**Pathogens:** *Candida* (9 BSI), Enterobacteriaceae (16 infections), MRSA, *Enterococcus*, *P. aeruginosa*|**Resistance phenotype:** Yes—MRSA in 5/23 (22%) VAP episodes|**Polymicrobial:** Yes (1 BSI; others not clearly specified)|**Risk factors for infection:** ECMO duration, transfused RBCs, SOFA score (multivariable: yes)|**Prophylaxis:** antibacterial—Not routine for ECMO; only after open-heart surgery or VAD; no SDD; antifungal—No antifungal prophylaxis|https://doi.org/10.1086/668439														
**Boscolo 2024 [22]**	Italy	Retro cohort (multicenter)	2017–2022	VV 100%	Respiratory/ARDS	279	54	72	NA	NR	NR	42 † (1-y)	N/NR	Narrative
**Case definition:** Primary exposure is MDR Gram-negative bacteria detected by routine surveillance, which includes colonization without clinical signs of infection. BSI/CR-BSI definitions are relegated to the supplement and not quantified in the main text. [MDR GN surveillance-based; BSI definitions in supplement]|**Infection sites:** VAP, BSI/CR-BSI, UTI, RTI|**Pathogens:** CRAB, DTR *K. pneumoniae*, DTR *P. aeruginosa*, ESBL, AmpC, CRE|**Resistance phenotype:** Yes—the study’s primary exposure. MDR GN 50% overall: 21% (*n* = 59) pre-ECMO, 29% (*n* = 80) acquired|**Polymicrobial:** Yes **Risk factors for infection:** Low ECMO volume center, interfacility transport, year of cannulation, SOFA score (multivariable: yes)|**Prophylaxis:** antibacterial—Antimicrobial prophylaxis at cannulation not administered; antifungal—NR|https://doi.org/10.1186/s13054-024-05068-x														
**Carelli 2023 [23]**	Italy	Prosp cohort	2020–2022	VV 100%	COVID-19 ARDS	68	53	82.4	A	44.1	13 (med)	60.3 (IH)	NR/NR	A, B
**Case definition:** De novo laboratory-confirmed BSI; ≥1 peripheral blood-culture set for a recognized pathogen; ≥2 peripheral sets for common skin commensals (CoNS, Corynebacterium, Bacillus); cultures drawn on clinical or biological signs of sepsis; primary vs. secondary BSI (same pathogen at another sterile site within 24 h) [LCBI-consistent (CDC/NHSN 2022 cited)]|**Infection sites:** BSI 30/68 (44%); VAP 59 (87%); UTI 17 (25%)|**Pathogens:** *E. faecalis*, *E. faecium*, MSSA, MRSA, *A. baumannii*, *P. aeruginosa*, *Klebsiella* spp., *E. coli*, *C. tropicalis*, *C. parapsilosis*|**Resistance phenotype:** Yes—MDR Gram-positive 4 and MDR Gram-negative 8 among first BSI events|**Polymicrobial:** Yes (some first BSI events; *n* NR)|**Risk factors for infection:** Shorter pre-ECMO respiratory support (OR 0.87, 0.77–0.99); longer ECMO duration (OR 1.06, 1.02–1.11) (multivariable: yes)|**Prophylaxis:** antibacterial—NR; antifungal—NR|https://doi.org/10.1016/j.hrtlng.2023.07.012														
**Cavayas 2018 [24]**	International (ELSO)	Registry (ELSO)	2006–2016	VV 58%, VA 35%, hybrid 6%	Mixed (respiratory)	2129 ^s^	48.5	59.7	NA	NA	NR	50.3 (IH)	NR/NR	Narrative
**Case definition:** ICD-9 coded fungal infection or any positive fungal culture [Other/unclassified]|**Infection sites:** BSI, pulmonary (esp. *Aspergillus*)|**Pathogens:** *Candida* spp., *Aspergillus* spp.|**Resistance phenotype:** NR|**Polymicrobial:** NR|**Risk factors for infection:** *Aspergillus*: transplant, influenza, respiratory ECMO; *Candida*: sepsis, RRT, age (multivariable: yes)|**Prophylaxis:** antibacterial—Registry does not capture prophylaxis; antifungal—Registry does not capture prophylaxis|https://doi.org/10.1186/s13054-018-2023-z														
**Gao 2023 [25]**	China	Retro cohort	2014–2022	VA 68%, VV 32%	Mixed	90	NR	76.7	NA	NR	NR	78.9 † (IH)	NR/NR	Narrative
**Case definition:** Cohort split into MDR-infection group (R) vs. non-MDR group (N). MDR identification per ‘proposed amendments regarding the definitions of MDR and XDR pathogens’. No BSI-specific case definition. [MDR-focused (Magiorakos); not a BSI case definition]|**Infection sites:** Respiratory secretions, blood, urine, drainage fluid, catheter blood|**Pathogens:** MDR Gram-negative bacteria (*A. baumannii*, *K. pneumoniae*, *P. aeruginosa*)|**Resistance phenotype:** Yes—the study’s primary exposure|**Polymicrobial:** NR|**Risk factors for infection:** Longer ventilator and ECMO duration, longer ICU/hospital stay (multivariable: yes)|**Prophylaxis:** antibacterial—NR; antifungal—NR|https://doi.org/10.2147/IDR.S421413														
**Garcia 2013 [26]**	International (ELSO)	Registry (ELSO)	1985–2009	VA 42%, VV 58% (adults)	Respiratory/ARDS	19 ^s^	30.2	47.4	NA	NA	8.2 † (med)	58 † (IH)	NR/NR	Narrative
**Case definition:** Aspergillus isolation; invasive in one case (autopsy) [Other/unclassified]|**Infection sites:** Respiratory/BSI (site unspecified)|**Pathogens:** *Aspergillus* spp.|**Resistance phenotype:** NR|**Polymicrobial:** NR|**Risk factors for infection:** No known immunosuppression in most (multivariable: no)|**Prophylaxis:** antibacterial—Registry; antifungal—Registry (fluconazole mention applies only to the infant case report)|https://doi.org/10.1177/0885066611432542														
**Grasselli 2017 [8]**	Italy	Retro (prosp data)	2010–2015	VV 87%, other 13% (ECCO_2_R 9%)	Mixed (respiratory)	92	48.5 ^a^ (med)	63	B	NR	14 (med)	31.5 (ICU)	NR/NR	A
**Case definition:** All positive cultures adjudicated by two intensivists and two ID specialists against ATS/IDSA 2005 (VAP), IDSA 2010 (CAUTI) and IDSA 2009 (catheter infection) guidelines; detailed criteria in the source report’s online supplement (not available); only the first NI episode analyzed [Guideline-based adjudication (ATS/IDSA)]|**Infection sites:** First NI: VAP 32 (35%), UTI 8 (9%), BSI 5 (5%), CRBSI 3 (3%), *C. difficile* colitis 2, cannula 1, PA catheter 1; 100 recurrent infections in 29 patients|**Pathogens:** *A. baumannii*, *Pseudomonas* spp., Enterobacteriaceae, *K. pneumoniae*, *Enterococcus* spp., *S. aureus*, CoNS, *Aspergillus* spp. (6), *Candida* spp. (3)|**Resistance phenotype:** Yes—MDR in 24 of 43 bacterial first NIs (56%; CDC definition)|**Polymicrobial:** NR|**Risk factors for infection:** Younger age (only independent factor, Cox); longer IMV > 3 d and non-VV setup non-significant (multivariable: yes)|**Prophylaxis:** antibacterial—Not described; all patients receiving antibiotics at ECMO start (infection at admission 71%); antifungal—Not described|https://doi.org/10.1097/CCM.0000000000002652														
**Haneke 2016 [19]**	Germany	Retro cohort	2008–2014	VV 76%, VA 9%, pECLA 19%	Mixed (trauma)	88	54 ^a^	85.2	B	NR	9.1 (med)	48.9 (IH)	N/N	Narrative
**Case definition:** CDC 1988 criteria [61]; infection > 48 h after ECMO initiation [CDC/NHSN]|**Infection sites:** Respiratory (69), BSI (40), UTI (5), others (skin, abdominal, CV)|**Pathogens:** *Staph. aureus*, *Enterococcus* spp., *Candida* spp., *K. pneumoniae*, *P. aeruginosa*, *Enterobacter* spp.|**Resistance phenotype:** Yes—MRSA 22% of 18 *S. aureus* isolates|**Polymicrobial:** Yes|**Risk factors for infection:** Not significant; nosocomial infections higher in survivors (longer stay) (multivariable: no)|**Prophylaxis:** antibacterial—No prophylactic antibiotics (ELSO-concordant); antifungal—Antimycotics therapeutic only|https://doi.org/10.1097/MAT.0000000000000308														
**Hsu 2009 [27]**	Taiwan	Retro cohort	2001–2007	VA 87%, VV 13%	Cardiogenic shock	114	51.3	67.5	B	2.6	8.8 † (mean)	75.4 † (IH)	NP/NR	A
**Case definition:** CDC/NHSN definitions (24 h post-ECMO to 48 h post-decannulation) [CDC/NHSN]|**Infection sites:** RTI (4), SSI (4), BSI (3), UTI (1)|**Pathogens:** *E. coli*, *P. aeruginosa*, *Burkholderia*, MRSA, *Candida*, VRE|**Resistance phenotype:** Yes—MRSA (2), VRE (1), ESBL-producing *E. coli*|**Polymicrobial:** Yes (some)|**Risk factors for infection:** Prolonged ECMO duration (>10 days) (multivariable: yes)|**Prophylaxis:** antibacterial—No defined regimen; 74.6% on antibiotics at ECMO day 1; antifungal—NR|https://doi.org/10.1016/j.jhin.2009.07.016														
**Joshi 2024 [28]**	India	Ambidirectional cohort	2021–2022	VV 83%, VV→VA 17%	COVID-19 ARDS	29	43	86.2	A	NR	61 (med)	44.8 (IH)	NR/NR	Narrative
**Case definition:** Surviving Sepsis Guidelines + NHSN + microbiological criteria [CDC/NHSN]|**Infection sites:** BSI (44.3%), Pneumonia (39%), SSTI (10.3%), CAUTI (3.8%), IAI (2.7%)|**Pathogens:** CRKP, *P. aeruginosa*, *A. baumannii*, *C. auris*, MDR GNB, *Enterococci*, others|**Resistance phenotype:** Yes—carbapenem-resistant *K. pneumoniae* (13/22 isolates; five also colistin-resistant); MDR/DTR and XDR *P. aeruginosa*; VRE (all *E. faecium*); *C. auris*|**Polymicrobial:** Yes|**Risk factors for infection:** Prolonged ECMO, immunosuppression (e.g., steroids, tocilizumab) (multivariable: no)|**Prophylaxis:** antibacterial—NR; antifungal—NR|https://doi.org/10.1016/j.ijmmb.2024.100539														
**Juthani 2018 [29]**	United States	Retro cohort	2012–2015	VV 64%, VA 36%	Mixed (respiratory)	100 ^u^	52	59	B	7.0	NR	38 † (NS)	N/NR	A
**Case definition:** CDC definition, >48 h after ECMO or ≤3 days post-decannulation [CDC/NHSN]|**Infection sites:** Respiratory (16), BSI (7), UTI (3)|**Pathogens:** Gram-negative (42.3%), Gram-positive (34.6%), Fungal (23.1%)|**Resistance phenotype:** NR|**Polymicrobial:** Yes|**Risk factors for infection:** ECMO >10 days, Black race, longer pre-ECMO stay (multivariable: yes)|**Prophylaxis:** antibacterial—Routine prophylaxis not practised; antifungal—NR|https://doi.org/10.1016/j.hrtlng.2018.07.004														
**Kang 2025 [30]**	China	Retro cohort	2021–2024	VA 59%, VV 41%	Mixed (NR)	59	53.1	61	C	20.3	NR	52.5 † (IH)	NR/NR	A
**Case definition:** No blood-culture-based CDC/NHSN criterion. Ascertainment conditional on temperature > 38.5 °C. [mNGS-based composite (not CDC/NHSN)]|**Infection sites:** BSI (20.3%), VAP (25.7%)|**Pathogens:** *A. baumannii*, *K. pneumoniae*, *S. epidermidis*, *S. aureus*, HHV, CMV, *Aspergillus*|**Resistance phenotype:** Partial—*A. baumannii* predominant; phenotype not systematically reported|**Polymicrobial:** Yes|**Risk factors for infection:** Diabetes, ECMO duration, lactate, GNB infection (multivariable: yes)|**Prophylaxis:** antibacterial—Not reported as prophylaxis (85% empirical therapy); antifungal—NR|https://doi.org/10.1155/ijcp/4985868														
**Kim 2016 [31]**	Republic of Korea	Retro cohort	2012–2014	VA 47%, VV 53%	Mixed (respiratory)	47 ^s^	53.1	61.7	B	27.7	12.2 † (mean)	38.3 † (IH)	NP/NR	A
**Case definition:** Positive blood culture > 48 h after ECMO initiation to 24 h post-decannulation [Institutional/culture-based]|**Infection sites:** BSI, catheter colonization|**Pathogens:** *A. baumannii*, *E. coli*, *P. aeruginosa*, *E. faecium*, *S. aureus*, *C. tropicalis*|**Resistance phenotype:** Yes—all catheter-colonizing organisms MDR (carbapenem-resistant *A. baumannii*, MRSA)|**Polymicrobial:** Yes|**Risk factors for infection:** Baseline sepsis, ECMO duration, BSI (multivariable: yes)|**Prophylaxis:** antibacterial—Empirical per guideline; no defined prophylaxis; antifungal—NR|https://doi.org/10.1007/s10047-015-0882-5														
**Kim 2017 [32]**	Republic of Korea	Retro cohort	2011–2015	VA 95%, VAV 5%	Cardiogenic shock	61	60.6	65.6	B	NR	6.8 (mean)	68.9 † (IH)	R/NR	A
**Case definition:** CDC/NHSN criteria; ≥48 h after ECMO until 24 h post-weaning [CDC/NHSN]|**Infection sites:** BSI (9), RTI (9)|**Pathogens:** *A. baumannii*, *P. aeruginosa*, *S. aureus*, *C. albicans* (no *Candida* BSI)|**Resistance phenotype:** NR|**Polymicrobial:** Yes (some cases)|**Risk factors for infection:** Pre-ECMO creatinine, ECMO duration (multivariable: yes)|**Prophylaxis:** antibacterial—Broad-spectrum at initiation (agent unspecified); antifungal—NR|https://doi.org/10.3346/jkms.2017.32.4.593														
**Kızmaz 2023 [33]**	Turkey	Retro cohort	2015–2023	VA 90%, VV 10%	Mixed (ECPR)	73	52.2	75.3	B	9.6	2 (med)	NR	S/NR	Narrative
**Case definition:** NI 24 h after initiation to 48 h after discontinuation; diagnosis per CDC 1988 definitions [61] (source ref. [13]) or the patient’s clinical status; not modern CDC/NHSN LCBI. [CDC 1988 (Garner/Hughes) + clinical status]|**Infection sites:** LRTI (47.3%), BSI (26.3%), CISI (15.7%), BSI + LRTI (5.2%)|**Pathogens:** *K. pneumoniae*, *B. cepacia, P. aeruginosa*, *E. cloacae*, *E. faecalis*, *S. maltophilia*, *A. baumannii*, *R. pickettii*, others|**Resistance phenotype:** No (*K. pneumoniae*, *E. faecalis, S. maltophilia* named; no resistance phenotype reported)|**Polymicrobial:** Yes|**Risk factors for infection:** ECMO duration > 3d, longer ICU/hospital stay, IABP, CRRT (multivariable: no)|**Prophylaxis:** antibacterial—Protocol: 1st-gen cephalosporin or glycopeptide; delivered in 21/73 (28.7%); antifungal—NR|https://doi.org/10.26355/eurrev_202308_33295														
**Ko 2020 [34]**	Republic of Korea	Retro cohort	2010–2018	VA 100%	ECPR (IHCA)	150	60	74.7	B	12	NR	50 † (IH)	NP/NR	A
**Case definition:** NIs defined per CDC definitions (source ref. [18]); only each patient’s first episode of NI included. [CDC]|**Infection sites:** Pneumonia (54%), CRBSI (9%), UTI (0.7%)|**Pathogens:** G- (*Klebsiella*, *Enterobacter*), G+ (*Staph* spp.), *Candida* spp.|**Resistance phenotype:** Yes—MDR NI in 10/35 (28.6%); MDR vs. non-MDR ICU mortality 80% vs. 32% (*p* = 0.028), in-hospital 82% vs. 46% (*p* < 0.001)|**Polymicrobial:** Yes (includes MDR)|**Risk factors for infection:** Low BMI, longer CPR to pump-on time (multivariable: yes)|**Prophylaxis:** antibacterial—No protocol; 82.7% received antibiotics on ECPR day; antifungal—NR|https://doi.org/10.1371/journal.pone.0243838														
**Lee 2022 [35]**	Republic of Korea	Retro cohort	2015–2021	VA, VV, VAV (% NR)	Mixed (cardiac)	480	56.3	65.4	B	23.5	NR	47.4 (IH)	NP/NP	A
**Case definition:** No CDC/NHSN citation. [Institutional/culture-based]|**Infection sites:** Bloodstream|**Pathogens:** MDR Gram-negative (*Acinetobacter*, *Klebsiella*), MR and MS CoNS, VRE, *Candida* spp.|**Resistance phenotype:** Yes—MDR Gram-negative (*Acinetobacter*, *Klebsiella*), methicillin-resistant CoNS, VRE|**Polymicrobial:** NR|**Risk factors for infection:** Hemodialysis, VAV mode, ECMO duration (multivariable: yes)|**Prophylaxis:** antibacterial—No routine protocol; 75% on antibiotics pre-ECMO; antifungal—No routine protocol; 13.7% on antifungals pre-ECMO|https://doi.org/10.1038/s41598-022-19405-z														
**Li 2023 [36]**	China	Retro cohort	2011–2020	VA 100%	Post-cardiotomy	503	61	71.4	B	19.9 ^e^	NR	51.9 (IH)	R/N	Narrative
**Case definition:** BSI = clinical manifestation (fever > 38 °C, chills or hypotension) + positive blood culture from one or more blood samples. No skin-commensal exclusion rule, which is more permissive than LCBI and may overestimate BSI. [China NHC 2001 (derived from CDC 1988 [61])—not CDC/NHSN LCBI]|**Infection sites:** VAP (30.2%), BSI (19.9%), UTI (0.8%), SSI/SSTI (8%)|**Pathogens:** *A. baumannii* (34%), *K. pneumoniae* (21.6%), *Enterobacter* spp., *S. aureus*, *Candida* spp.|**Resistance phenotype:** Yes 1|**Polymicrobial:** Yes (not quantified)|**Risk factors for infection:** CRRT, VIS > 40, SOFA score, bleeding, cerebral complication (multivariable: yes)|**Prophylaxis:** antibacterial—All patients at VA-ECMO initiation; ceftazidime + vancomycin most common; antifungal—Antifungals only if fungal infection suspected|https://doi.org/10.2147/IDR.S390599														
**Menaker 2019 [37]**	United States	Retro cohort	2010–2015	VV 54%, VA 46%	Mixed	268	NR	61.2	B	9.7	NR	46.4 † (IH)	S/NR	A
**Case definition:** CDC/NHSN criteria: 2+ positive cultures + symptoms [CDC/NHSN]|**Infection sites:** BSI only|**Pathogens:** *Candida* spp, GNR (e.g., *Pseudomonas*, *Acinetobacter*), *Enterococcus*, *Streptococcus*|**Resistance phenotype:** NR (MRSA/MDR-GNB surveillance described; isolate phenotypes not reported)|**Polymicrobial:** Yes|**Risk factors for infection:** ECMO duration, RBC/PLT transfusion, renal/hepatic failure (multivariable: yes)|**Prophylaxis:** antibacterial—Mode-specific: VA peripheral vancomycin + ceftriaxone 48 h; VA central/open sternum vancomycin + cefazolin to 48 h post-closure; VA in ICU vancomycin + pip/tazo; VV none; antifungal—None stated|https://doi.org/10.1016/j.hrtlng.2019.01.004														
**Na 2018 [38]**	Republic of Korea	Retro cohort	2012–2016	VV 100%	Respiratory/ARDS	121	60	72.7	A	17.4	14 (med)	61.2 † (IH)	NP/NP	A, B
**Case definition:** LCBI criteria: recognized pathogen from blood culture, OR clinical manifestation + skin commensal from ≥2 cultures. Window > 48 h after initiation to decannulation. [CDC/NHSN]|**Infection sites:** BSI (17%), RTI (source in 62% of secondary BSI), UTI, intra-abdominal|**Pathogens:** *S. aureus*, *A. baumannii*, *Candida tropicalis*, *C. albicans*, others|**Resistance phenotype:** No (*S. aureus*, *A. baumannii*, *S. maltophilia*, *P. aeruginosa* named; no phenotype)|**Polymicrobial:** Yes|**Risk factors for infection:** Duration of arterial catheterization, blood transfusion volume (multivariable: yes)|**Prophylaxis:** antibacterial—No prophylaxis protocol (92% broad-spectrum therapy); antifungal—No protocol (27% received antifungals)|https://doi.org/10.1017/ice.2018.90														
**Nesseler 2024 [39]**	France	Prosp cohort (multicenter)	2020–2022	VV 91%, VA 9%	COVID-19 ARDS	602	NR	77.6	A	25.6	NR	56.6 (IH)	NP/NR	A, B
**Case definition:** BSI = positive blood culture ≥ 48 h after admission; skin commensals require two cultures from separate puncture sites. Cites the EUROBACT criteria (Tabah et al.; source ref. [14]), not CDC/NHSN. [EUROBACT/LCBI-equivalent (not a CDC/NHSN citation)]|**Infection sites:** BSI (26%), VAP (19%); 27% had both|**Pathogens:** Enterobacteriaceae, *Enterococcus* spp., non-fermenting GNB, MRSA, fungi|**Resistance phenotype:** Yes—MDRO 21% of BSI, 15% of VAP; ESBL-PE 3.9%; MRSA 2.6%; VRE 0.6%; imipenem-R Acinetobacter 0.6%|**Polymicrobial:** Yes (20% BSI, 14% VAP)|**Risk factors for infection:** Male sex, chronic disease, SOFA score, center experience, antibiotic use (multivariable: yes)|**Prophylaxis:** antibacterial—No common protocol; SDD in 15/602 (2.5%); antifungal—NR|https://doi.org/10.1186/s13054-024-04832-3														
**Ortuño 2025 [40]**	France/Europe	Retro case series (multicenter)	2012–2022	VA 71%, VV 29%	Mixed (cardiogenic shock)	109 ^s^	52	68	NA	NA	15 (med)	51 ^s^ (ICU)	NR/NR	Narrative
**Case definition:** ECMO-CRI = clinical suspicion plus a positive bacterial sample from the ECMO cannulation site. [ECMO cannula-related infection (ECMO-CRI)]|**Infection sites:** Cannula insertion site|**Pathogens:** Enterobacteriaceae (51%), CoNS (29%), *Enterococcus* spp. (30%), polymicrobial (39%)|**Resistance phenotype:** NR|**Polymicrobial:** Yes|**Risk factors for infection:** BSI and septic shock associated with increased ICU mortality (multivariable: yes)|**Prophylaxis:** antibacterial—Center procedures in the online supplement (not accessed); antifungal—Online supplement (not accessed)|https://doi.org/10.1186/s13613-025-01446-y														
**Pieri 2012 [41]**	Italy	Case-control	2009–2011	VA 59%, VV 41%	Mixed	27	54.9	74.1	A	11.1	6.6 (mean)	29.6 (on ECMO)	R/N	B
**Case definition:** CDC criteria + microbiology [CDC/NHSN]|**Infection sites:** BSI (3), Pneumonia (8), Cholecystitis (1), Unknown (2)|**Pathogens:** *Candida albicans*, *E. coli*, *K. pneumoniae*, MRSA, *A. baumannii*, others|**Resistance phenotype:** Yes—MRSA (1 pneumonia)|**Polymicrobial:** NR|**Risk factors for infection:** Age (inverse), pre-ECMO infection, ECMO duration, biomarker combo (PCT + CRP) (multivariable: yes)|**Prophylaxis:** antibacterial—Cefazolin 48 h after cannulation; antifungal—No antifungal prophylaxis|https://doi.org/10.1016/j.jtcvs.2012.01.005														
**Pieri 2013 [42]**	Italy	Retro cohort	2009–2011	VA 52%, VV 48% (ECMO subset)	Mixed	46	NR	NR	B	17.4	NR	NR	R/N	B(s)
**Case definition:** Positive culture from any site (blood, BAL, urine). No BSI-specific criteria. [Institutional/culture-based]|**Infection sites:** Pneumonia, bloodstream, surgical site, urinary tract|**Pathogens:** *Candida* spp. (29%), *Klebsiella* spp. (21%), Gram-negative and positive bacteria, fungi|**Resistance phenotype:** NR|**Polymicrobial:** Yes (22% in ECMO group)|**Risk factors for infection:** SOFA score at implant, VAD support (multivariable: yes)|**Prophylaxis:** antibacterial—Cefazolin 48 h after cannulation; antifungal—None during study (policy changed afterwards)|https://doi.org/10.1016/j.medin.2012.08.009														
**Pluim 2012[43]**	International (ELSO)	Registry (ELSO)	1997–2009	VA 61%, VV 39% (adults)	Mixed (NR)	2067	44.6	60.3	NA	NR	NR	NR	NR/NR	A(s)
**Case definition:** Registry infection coding, all ages; fungal-timing strata. [ELSO registry-coded]|**Infection sites:** Not specified (fungal infection coded generically)|**Pathogens:** *Candida* spp. (majority), non-*Candida* fungi|**Resistance phenotype:** NR|**Polymicrobial:** NR|**Risk factors for infection:** Age, support type, ECMO duration (multivariable: yes)|**Prophylaxis:** antibacterial—Registry; antifungal—Registry|https://doi.org/10.1097/PCC.0b013e31824fbaf7														
**Poth 2022 [44]**	Germany	Retro cohort + PS-matched comparison	2013–2021	VV 100%	Respiratory/ARDS	368	NR	NR	NA	5.4 ^f^	NR	NR	NR/N	A(s)
**Case definition:** *Candida* from sterile sites = proven IFD (EORTC/MSG 2020); *Candida* in tracheal aspirate, BAL or urine = colonization; Aspergillus = any culture from respiratory fluids with clinical features (does not meet consensus criteria) [Consensus criteria (fungal)]|**Infection sites:** Candidemia 20/368 (5.4%); invasive aspergillosis (BALF) 2/368 (0.5%)|**Pathogens:** *C. albicans* 15, *C. krusei* 2, *C. glabrata* 1, *C. kefyr* 1, *C. parapsilosis* 1, *A. terreus* 1, *A. fumigatus* 1|**Resistance phenotype:** Antifungal susceptibility reported: all isolates susceptible to the initial agent (EUCAST)|**Polymicrobial:** NR|**Risk factors for infection:** Descriptive only (IFD group): TPN 72%, broad-spectrum antibiotics > 2 weeks 68% (multivariable: no)|**Prophylaxis:** antibacterial—NR; antifungal—No prophylaxis or pre-emptive therapy for candidiasis during the study period|https://doi.org/10.3390/jcm11071940														
**Poth 2024 [45]**	Germany	Retro cohort	2013–2023	VA 100%	Cardiogenic shock	392	64.2	72.7	NA	3.8 ^f^	NR	68.6 † (IH)	NR/N	A
**Case definition:** CDC/EORTC/ESICM consensus, IC & pIPA criteria applied [Consensus criteria (fungal)]|**Infection sites:** BSI (all), mediastinum, pericardium, LVAD; pIPA (1.0%)|**Pathogens:** *Candida albicans*, *C. glabrata*, *C. parapsilosis*, *Aspergillus fumigatus*|**Resistance phenotype:** No—all fungal isolates susceptible to first-line antifungal (EUCAST)|**Polymicrobial:** Yes (22%)|**Risk factors for infection:** SAPS II score, CKRT before IFD (multivariable: yes)|**Prophylaxis:** antibacterial—NR; antifungal—Standardized antifungal prophylaxis not performed|https://doi.org/10.1016/j.jcrc.2024.154831														
**Quintana 2021 [46]**	United States	Retro cohort	2015–2017	VA 72%, VV 26%, VAV 2%	Mixed (cardiac)	288	NR	59.4	B	7.6	NR	37.5 † (IH)	R/NR	A
**Case definition:** ‘Bloodstream infection was defined by positive blood cultures with a pathogenic organism.’ No commensal two-culture rule. CLABSI is defined separately and idiosyncratically (‘within 48 h of central line placement’). [Institutional/culture-based (not CDC/NHSN)]|**Infection sites:** Pneumonia (11.7%), BSI (7.6%), UTI (1.4%)|**Pathogens:** *E. coli*, *Klebsiella*, *Candida*, *Enterococcus, S. aureus*, *Pseudomonas*|**Resistance phenotype:** NR|**Polymicrobial:** Yes (including mixed bacteremia + pneumonia)|**Risk factors for infection:** CRRT, CKD, ECMO duration, not cannulation site (multivariable: yes)|**Prophylaxis:** antibacterial—Vancomycin + ceftriaxone 48 h (vancomycin + ciprofloxacin if allergic); open chest to 48 h post-closure; antifungal—None stated|https://doi.org/10.1016/j.athoracsur.2020.12.012														
**Schmidt 2012 [10]**	France	Retro cohort	2003–2009	VA 100%	Cardiogenic shock	220	48.9	66.8	A	17.7	13.4 † (mean)	45 † (NS)	S/NR	A, B
**Case definition:** CDC NNIS definitions (Garner 1988 [61]); ECMO-associated = onset >24 h after ECMO initiation to 48 h after discontinuation; BSI catheter-related when the same organism is recovered from blood and catheter tip; >2 positive blood cultures required for coagulase-negative staphylococci; blood cultures drawn for temperature > 38 °C or <36 °C or signs of sepsis [CDC/NHSN (NNIS 1988)]|**Infection sites:** Any NI 142/220 (64.5%); VAP 120 (55%; 163 episodes); BSI 39 (18%; 47 episodes); mediastinitis 20; cannula infection 20; catheter-related 10; UTI not surveyed|**Pathogens:** BSI: *P. aeruginosa* 10, *Enterococcus* spp. 7, *E. coli* 6, *S. epidermidis* 5, *S. aureus* 4, *Streptococcus* 3, *Enterobacter* 3, *Candida* 3, anaerobes 3; VAP: *P. aeruginosa*, *S. aureus*, *Enterobacter*, *E. coli*, *H. influenzae*, *Klebsiella*|**Resistance phenotype:** NR|**Polymicrobial:** Yes (VAP 19 episodes; BSI 1 episode)|**Risk factors for infection:** SOFA at ICU admission (HR 1.04, 1.00–1.08); antibiotics at cannulation not protective (HR 0.73, 0.50–1.05) (multivariable: yes)|**Prophylaxis:** antibacterial—Single IV dose of second-generation cephalosporin or glycopeptide only for intrathoracic cannulation in patients not already on antibiotics; no SDD; antifungal—NR|https://doi.org/10.1093/cid/cis783														
**Schweickhardt 2023 [47]**	United States	Retro cohort	2013–2021	VV 91%, VA 9%	Trauma	21 ^s^	30	85.7	NA	NA	9.8 (med)	24 ^s^ (IH)	N/N	Narrative
**Case definition:** Positive culture + treated with antibiotics [Institutional/culture-based]|**Infection sites:** Respiratory (13), SSTI (6), BSI (4), UTI (1)|**Pathogens:** *Pseudomonas*, *Klebsiella*, *Acinetobacter*, *Enterobacter*, MDR-GNB, *Candida*|**Resistance phenotype:** Yes—MDR organisms 9/26 isolates (35%; ≥3 antibiotic classes)|**Polymicrobial:** Yes|**Risk factors for infection:** Trauma, early cannulation, abdominal injuries (multivariable: no)|**Prophylaxis:** antibacterial—No prophylactic antibiotics for ECMO; antifungal—No prophylactic antifungals|https://doi.org/10.1016/j.injury.2022.11.048														
**Selçuk 2021 [48]**	Turkey	Retro cohort	2012–2016	VA 97%, VV 3% (whole cohort)	Post-cardiotomy	60 ^b^	54.9	66.7	B	3.3	7.9 (mean)	58.3 (NS)	N/N	A
**Case definition:** CDC 1988 (Garner) [61] nosocomial infection criteria; ECMO-related infection = onset from 24 h after ECMO initiation to 48 h after ECMO end; no commensal rule stated [CDC 1988 (Garner)]|**Infection sites:** Adults: LRTI 10 (16.7%), surgical wound 8 (13.3%), catheter wound 5 (8.3%), BSI 2 (3.3%), UTI 2 (3.3%); one site per infected patient|**Pathogens:** Adults: *Klebsiella* 6, *Streptococcus* 3, *Acinetobacter* 3, *E. cloacae* 3, CoNS 2, *E. coli* 2, *Pseudomonas* 2, *Serratia* 2; no *Candida*|**Resistance phenotype:** NR|**Polymicrobial:** NR|**Risk factors for infection:** Hemodialysis, ECMO > 5 days (univariate only) (multivariable: no)|**Prophylaxis:** antibacterial—No prophylaxis except surgical prophylaxis on the day of surgery; antifungal—No prophylaxis (institutional policy)|https://doi.org/10.21470/1678-9741-2020-0077														
**Silvetti 2019 [49]**	Italy	Retro cohort	2013–2017	VA 100%	Post-cardiotomy	31 ^b^	NR	NR	B	22.6	NR	71 † (IH)	R/NR	A
**Case definition:** New positive blood culture 48 h after ECMO initiation to 48 h after discontinuation. Not CDC/NHSN. [Institutional/culture-based]|**Infection sites:** BSI (100%), VAP (71%), UTI (21%), catheter/SSTI (7%)|**Pathogens:** *P. aeruginosa* (26.6%), *K. pneumoniae* (20%), *Candida* (1 pt), Gram+ (40%)|**Resistance phenotype:** No (GN 53%: *P. aeruginosa* 26.6%, *K. pneumoniae* 20%; GP 40%; *Candida* 1)—no MDR phenotype|**Polymicrobial:** Yes (28% had co-infections)|**Risk factors for infection:** Adult age, pre-ECMO bilirubin, chest drain blood loss (multivariable: yes)|**Prophylaxis:** antibacterial—Open chest: ceftazidime + vancomycin + amikacin; closed chest: perioperative ceftazidime; antifungal—NR|https://doi.org/10.1177/0391398818817325														
**Sun 2010 [11]**	Taiwan	Retro cohort	1996–2007	VA 80%, VV 17%	Mixed (cardiac)	334	51 ^a^	68	B	10.2	7.7 † (mean)	68.3 (IH)	R/NR	A, B(s)
**Case definition:** CDC criteria; nosocomial during ECMO only [CDC/NHSN]|**Infection sites:** BSI (38), SSI (6), RTI (4), UTI (3), SSTI (2), Catheter (2)|**Pathogens:** *S. maltophilia*, *Candida* spp., *P. aeruginosa*, *K. pneumoniae*, *E. coli*, *S. aureus*|**Resistance phenotype:** Yes—oxacillin-resistant S. aureus 6/7 and CoNS 3/3; carbapenem-nonsusceptible *A. baumannii* 3/5 and *P. aeruginosa* 2/5|**Polymicrobial:** NR|**Risk factors for infection:** VV mode, autoimmune disease, mechanical complications, longer ECMO duration (multivariable: yes)|**Prophylaxis:** antibacterial—Ceftazidime + vancomycin at initiation if not already on antibiotics; antifungal—None stated|https://doi.org/10.1016/j.jtcvs.2010.07.017														
**Suzuki 2023 [50]**	International (ELSO)	Registry (ELSO)	2000–2019	VA/VV (% NR)	Oncologic (mixed)	1871 ^u^	NR	NR	NA	NR	NR	NR	NR/NR	Narrative
**Case definition:** Culture-proven infection during ECMO [Institutional/culture-based]|**Infection sites:** Pulmonary (most common), others NR|**Pathogens:** *Klebsiella*, *Candida*, CoNS (no detailed frequencies)|**Resistance phenotype:** NR|**Polymicrobial:** NR|**Risk factors for infection:** Adult age, ARDS indication, longer ECMO duration (multivariable: yes)|**Prophylaxis:** antibacterial—NR; antifungal—NR|https://doi.org/10.1177/08850666221128243														
**Sysiak-Sławecka 2023 [51]**	Poland	Retro cohort	2021	VV 100%	COVID-19 ARDS	15	40	53.3	NA	40.0 ^c^	NR	67 (NS)	N/N	Narrative
**Case definition:** New infection (bacteremia, VAP, UTI) during ICU stay, clinical and culture-based [Institutional/culture-based]|**Infection sites:** BSI, VAP, UTI (proportions detailed)|**Pathogens:** *A. baumannii*, *K. pneumoniae* (ESBL+, MBL+), E. coli, MRSA, MSSA, *E. faecalis*, *S. epidermidis*, *S. anginosus*, *P. aeruginosa*|**Resistance phenotype:** Yes—ESBL/MBL-producing *K. pneumoniae*, MRSA, HLAR *E. faecium*, AmpC *S. marcescens*|**Polymicrobial:** Yes (not quantified)|**Risk factors for infection:** Higher urea associated with superinfection (OR 0.942) (multivariable: yes)|**Prophylaxis:** antibacterial—Departmental policy: no antimicrobial prophylaxis during MV/VV-ECMO; antifungal—Policy covers all antimicrobials|https://doi.org/10.5114/ait.2023.130833														
**Thomas 2017 [52]**	France	Prosp cohort	34 mo (dates NR)	VV 100%	Respiratory/ARDS	103 ^u^	49	64.1	A	19.4	11 (med)	56.3 (IH)	N/NR	Narrative
**Case definition:** CDC and French catheter infection guidelines adapted for ECMO [CDC/NHSN]|**Infection sites:** Cannula and bloodstream|**Pathogens:** CoNS (80% of infections, 60.6% colonization)|**Resistance phenotype:** NR|**Polymicrobial:** NR|**Risk factors for infection:** None identified (multivariable: yes)|**Prophylaxis:** antibacterial—No specific antibioprophylaxis at cannulation; antifungal—NR|https://doi.org/10.1186/s13613-017-0335-9														
**Uçar 2024 [53]**	Turkey	Retro cohort	2018–2021	VV 100%	Respiratory/ARDS	50	46	76	A	54.0	16.6 (mean)	80 (ICU)	NR/NR	B
**Case definition:** CDC/NHSN (Horan 2008); ECMO-related = diagnosed after the first 48 h of cannulation up to 48 h after decannulation; blood cultures from two different sites on fever ≥ 37.5 °C, leukocytosis or raised CRP/PCT [CDC/NHSN]|**Infection sites:** Any NI 30 (60%); BSI 27 (54%); VAP 28 (56%); catheter-related UTI 14 (28%)|**Pathogens:** BSI (46 isolates): *K. pneumoniae* 10, *A. baumannii* 7, *S. maltophilia* 7, CoNS 6, *P. aeruginosa* 4, *C. albicans* 4, *E. faecalis* 3, *Enterobacter* 2, *B. cepacia* 2, *E. coli* 1|**Resistance phenotype:** Yes—68/117 isolates MDR (58%); 8 PDR (7%) (Magiorakos)|**Polymicrobial:** NR **Risk factors for infection:** Multivariable: prior antibiotic use protective (OR 0.14, 0.03–0.70); ECMO duration (OR 1.13, 1.03–1.23). Univariate: COVID-19, steroids (multivariable: yes)|**Prophylaxis:** antibacterial—Ambiguous: prophylactic antibiotics “not used routinely” in patients already on antibiotics; practice in antibiotic-naive patients not stated; antifungal—NR|https://doi.org/10.1177/02676591231194931														
**Wang 2020 [54]**	China	Retro cohort	2013–2019	VA 84%, VV 16%	Cardiogenic shock	69	42	62.3	B	27.5	6.4 † (med)	40.6 † (IH)	R/NR	A
**Case definition:** Garner 1988 CDC definitions [61] (source ref. [7]), cited only for the nosocomial pneumonia definition. No BSI/LCBI criteria stated anywhere. [CDC 1988 (Garner) implicit—no explicit BSI criteria]|**Infection sites:** BSI (19), VAP (14), UTI (10)|**Pathogens:** *K. pneumoniae*, *E. aerogenes, A. baumannii*, *B. cepacia*, *P. aeruginosa*, *E. cloacae*, *S. marcescens, S. maltophilia*, *S. epidermidis*, *E. faecium*, *Candida albicans*, *C. parapsilosis*|**Resistance phenotype:** Yes—MRSA (higher proportion in the BSI group)|**Polymicrobial:** Yes|**Risk factors for infection:** Pre-ECMO SOFA, MRSA prophylaxis, CRRT, ventilator duration before ECMO weaning (multivariable: yes)|**Prophylaxis:** antibacterial—All patients during ECMO (pip/tazo most common; anti-MRSA combination 23/69); antifungal—NR|https://doi.org/10.12669/pjms.36.6.2882														
**Wang 2021 [55]**	China	Retro cohort	2012–2017	VA 100%	Post-cardiotomy	322	57.2	74.2	B	12.4 ^e^	5.2 † (mean)	50.9 † (IH)	R/NR	A
**Case definition:** CDC definitions; occurring ≥ 24 h after ECMO initiation and ≤48 h post-decannulation [CDC/NHSN]|**Infection sites:** RTI (76), BSI (40), SWI (17), UTI (10)|**Pathogens:** *A. baumannii*, *P. aeruginosa*, *K. pneumoniae*, *Enterobacter*, CoNS, *Candida* spp., *Micrococcus* spp.|**Resistance phenotype:** NR|**Polymicrobial:** Yes (not quantified)|**Risk factors for infection:** BMI, ECMO > 92 h, surgical time, CPB assist (multivariable: yes)|**Prophylaxis:** antibacterial—2nd-generation cephalosporin at initiation; antifungal NR|https://doi.org/10.21470/1678-9741-2020-0068														
**Wang 2023 [18]**	China	Retro cohort (multicenter)	2015–2021	VA 66%, VV 34%	Mixed	196	56.5 ^a^	70.4	NA	NR	NR	NR	NR/NR	Narrative
**Case definition:** ≥24 h post-ECMO initiation to ≤48–72 h post-weaning (RT-NISS + clinician confirmation) [Institutional/culture-based]|**Infection sites:** HAP (59), BSI (36), UTI (10), SSTI (5)|**Pathogens:** *A. baumannii*, *K. pneumoniae*, *P. aeruginosa*, *S. maltophilia*, MRSA, *Candida* spp.|**Resistance phenotype:** Yes—all *A. baumannii* MDR (100% carbapenem-resistant); *K. pneumoniae* MDR 18/20 (90%); MRSA|**Polymicrobial:** Yes (multiple co-pathogens)|**Risk factors for infection:** Pre-ECMO IMV, ECMO duration (multivariable: yes)|**Prophylaxis:** antibacterial—NR; antifungal—NR|https://doi.org/10.1016/j.ajic.2023.04.010														
**Wells 2025 [56]**	United States	Retro cohort	2022–2024	VV 62%, VA 15%, V-P 23%	Mixed (respiratory)	68	NR	NR	NA	NA	NR	NR	N/NR	Narrative
**Case definition:** The paper’s purpose is definitional: it proposes that CoNS bacteremia require isolation from two subsequent blood cultures. Only 5/13 (38%) met that bar. [CoNS-only ascertainment; study proposes a definition]|**Infection sites:** BSI only (CoNS)|**Pathogens:** *S. epidermidis* (80%), *S. hominis* (13%), *S. capitis* (7%)|**Resistance phenotype:** NR|**Polymicrobial:** No (all monomicrobial)|**Risk factors for infection:** Not statistically associated (e.g., fever, leukocytosis) (multivariable: no)|**Prophylaxis:** antibacterial—Center does not use prophylactic antibiotics; antifungal—NR|https://doi.org/10.1016/j.ajic.2025.02.004														
**Xu 2022 [57]**	China	Retro cohort	2011–2020	VV 77%, VA 23%	Respiratory/ARDS	79	53.3	66	A	24.1 ^e^	15.9 (mean)	68 (ICU)	N/N	A
**Case definition:** BSI = ≥1 positive blood culture for an identified pathogen, OR two positive cultures within 48 h for a common skin contaminant plus symptoms. LCBI-consistent. [CDC/NHSN]|**Infection sites:** VAP (30), BSI (19), UTI (4|**Pathogens:** *A. baumannii*, *K. pneumoniae*, *P. aeruginosa*, *S. aureus*, *Burkholderia*, *Candida*|**Resistance phenotype:** Yes—MDR defined per Magiorakos (resistance to ≥1 agent in ≥3 categories)|**Polymicrobial:** Yes|**Risk factors for infection:** ECMO duration, viral pneumonia, older age, transfusion, VV ECMO (multivariable: yes)|**Prophylaxis:** antibacterial—No prophylactic antimicrobial therapy after connection; antifungal—Statement covers all antimicrobials|https://doi.org/10.2147/IDR.S372913														
**Yang 2022 [58]**	China	Retro cohort	2013–2019	VV 100%	Respiratory/ARDS	105	49	72.4	A	21.9	11 (med)	63.8 † (IH)	N/NR	A, B
**Case definition:** Explicitly per CDC/NHSN definitions: two separate blood cultures positive for the same organism + signs of infection; named skin commensals require two cultures within 48 h. Window 24 h after ECMO to 48 h after withdrawal. [CDC/NHSN]|**Infection sites:** BSI only|**Pathogens:** *A. baumannii*, *Burkholderia cepacia*, *P. aeruginosa*, *K. pneumoniae*, *E. faecium, Candida*|**Resistance phenotype:** Yes—CRE in 2/3 K. pneumoniae; sulfamethoxazole-trimethoprim resistance 81.8%|**Polymicrobial:** NR|**Risk factors for infection:** Male sex, HTN, high Murray score, transfusion, CRRT (multivariable: yes)|**Prophylaxis:** antibacterial—No antibiotic prophylaxis; antifungal—NR|https://doi.org/10.1186/s12890-022-02164-y														
**Yun 2021 [59]**	Republic of Korea	Retro cohort	2009–2016	VA 54%, VV 46%	Mixed (NR)	1100	NR	NR	B	5.5	7.1 † (mean)	NR	NR/NR	Narrative
**Case definition:** BSI 2 d after ECMO initiation to 2 d after removal [Institutional/culture-based]|**Infection sites:** BSI (100%); sources: CRI (38%), pneumonia (31%)|**Pathogens:** *Enterococcus* spp., CoNS, *A. baumannii*, *K. pneumoniae*, *Candida* spp., others|**Resistance phenotype:** Yes—VRE 7/12 (58%) enterococci; carbapenem-R *A. baumannii* 9/10 (90%); ESBL *K. pneumoniae* 4/8 (50%); CRKP 2/8 (25%); methicillin-R CoNS|**Polymicrobial:** Yes (8 episodes)|**Risk factors for infection:** Gram-negative rods BSI, age, APACHE II score (multivariable: yes)|**Prophylaxis:** antibacterial—NR; antifungal—NR|https://doi.org/10.1177/0885066620985538														
**Zhao 2025 [60]**	China	Retro cohort	2017–2023	VA/VV (% NR)	Mixed (ECPR)	186	NR	NR	B	NR	NR	NR	R/N	Narrative
**Case definition:** Definitions referenced to Spalding/Zingg conventions; BSI not separately quantified at patient level. [CDC/NHSN-referenced]|**Infection sites:** Respiratory (75%), BSI (55%), UTI (14%)|**Pathogens:** *A. baumannii* (46.4%), *K. pneumoniae* (35.7%), *S. aureus*, *Candida* spp.|**Resistance phenotype:** Partial—*A. baumannii* and *K. pneumoniae* predominant; phenotype not systematically reported|**Polymicrobial:** Yes (32.1%)|**Risk factors for infection:** Recent antibiotics, steroids/immunosuppressants, IL-6, hs-CRP, thrombocytopenia (multivariable: yes)|**Prophylaxis:** antibacterial—1st-generation cephalosporin before cannulation, 24–48 h; antifungal—No antifungal prophylaxis|https://doi.org/10.3389/fmicb.2025.1555701														

Notes. Values are as reported unless marked †, derived by the review team from reported counts or strata. NR, not reported; NA, not applicable by design. BSI tiers are defined in Section 2.7. Prophylaxis (antibacterial/antifungal): R, routine; S, selective; NP, no protocol; N, no prophylaxis. Pooled role: A, Outcome A primary analysis (k = 24); B, Outcome B primary analysis (k = 7); (s), sensitivity analysis only; Narrative, narrative synthesis only. ^u^ Unit of analysis is ECMO runs or procedures, not patients. ^s^ Selected sample: sampled on infection status, so BSI prevalence is NA and mortality refers to the selected sample; Kim et al. [31] restricted their sample to patients with an ECMO catheter-tip culture. ^a^ Adult threshold below 18 years, or cohort may include patients younger than 18 years. ^b^ Adult subgroup of a mixed-age cohort. ^e^ BSI count episode-based or possibly overlapping. ^f^ Candidemia only. ^c^ Positive blood culture at any time during the ICU stay, not restricted to ECMO support. Abbreviations. AB, antibacterial; AF, antifungal; AmpC, AmpC β-lactamase; APACHE, Acute Physiology and Chronic Health Evaluation; ARDS, acute respiratory distress syndrome; ATS/IDSA, American Thoracic Society/Infectious Diseases Society of America; BAL, bronchoalveolar lavage; BALF, bronchoalveolar lavage fluid; BMI, body mass index; BSI, bloodstream infection; CAPA, COVID-19-associated pulmonary aspergillosis; CAUTI, catheter-associated urinary tract infection; CDC, Centers for Disease Control and Prevention; CISI, cannula insertion-site infection; CKD, chronic kidney disease; CKRT, continuous kidney replacement therapy; CLABSI, central line-associated bloodstream infection; CMV, cytomegalovirus; CoNS, coagulase-negative staphylococci; COVID-19, coronavirus disease 2019; CPB, cardiopulmonary bypass; CPR, cardiopulmonary resuscitation; CR-BSI/CRBSI, catheter-related BSI; CRAB, carbapenem-resistant Acinetobacter baumannii; CRE, carbapenem-resistant Enterobacterales; CRI, cannula-related infection; CRKP, carbapenem-resistant Klebsiella pneumoniae; CRP, C-reactive protein; CRRT, continuous renal replacement therapy; CV, cardiovascular; DOI, digital object identifier; DTR, difficult-to-treat resistance; ECCO_2_R, extracorporeal carbon dioxide removal; ECMO, extracorporeal membrane oxygenation; ECPR, extracorporeal cardiopulmonary resuscitation; ELSO, Extracorporeal Life Support Organization; EORTC/MSG, European Organisation for Research and Treatment of Cancer/Mycoses Study Group; ESBL, extended-spectrum β-lactamase; ESBL-PE, ESBL-producing Enterobacterales; ESICM, European Society of Intensive Care Medicine; EUCAST, European Committee on Antimicrobial Susceptibility Testing; EUROBACT, European bacteremia study group criteria; GN/GNB, Gram-negative (bacilli); GNR, Gram-negative rods; GP, Gram-positive; HAP, hospital-acquired pneumonia; HHV, human herpesvirus; HLAR, high-level aminoglycoside resistance; HR, hazard ratio; HSV, herpes simplex virus; HTN, hypertension; IABP, intra-aortic balloon pump; IAI, intra-abdominal infection; IC, invasive candidiasis; ICD-9, International Classification of Diseases, 9th revision; ICU, intensive care unit; ID, infectious diseases; IFD, invasive fungal disease; IH, in-hospital (death before hospital discharge); IHCA, in-hospital cardiac arrest; IL-6, interleukin 6; IMV, invasive mechanical ventilation; IV, intravenous; LCBI, laboratory-confirmed bloodstream infection; LRTI, lower respiratory tract infection; LVAD, left ventricular assist device; MBL, metallo-β-lactamase; MDR, multidrug-resistant; MDRO, multidrug-resistant organism; med, median; mNGS, metagenomic next-generation sequencing; MR/MS, methicillin-resistant/-susceptible; MRSA/MSSA, methicillin-resistant/-susceptible Staphylococcus aureus; MV, mechanical ventilation; NHC, National Health Commission (China); NHSN, National Healthcare Safety Network; NI, nosocomial infection; NNIS, National Nosocomial Infections Surveillance; NS, endpoint not stated; OR, odds ratio; PA, pulmonary artery; PCT, procalcitonin; PDR, pandrug-resistant; pECLA, pumpless extracorporeal lung assist; pip/tazo, piperacillin/tazobactam; pIPA, putative invasive pulmonary aspergillosis; PLT, platelet; Prosp, prospective; PS, propensity score; RBC, red blood cell; Retro, retrospective; RRT, renal replacement therapy; RT-NISS, real-time nosocomial infection surveillance system; RTI, respiratory tract infection; SAPS, Simplified Acute Physiology Score; SDD, selective digestive decontamination; SOFA, Sequential Organ Failure Assessment; SSI, surgical site infection; SSTI, skin and soft-tissue infection; SWI, surgical wound infection; TPN, total parenteral nutrition; UTI, urinary tract infection; VA, veno-arterial; VAD, ventricular assist device; VAP, ventilator-associated pneumonia; VAV, veno-arteriovenous; VIS, vasoactive-inotropic score; V-P, veno-pulmonary; VRE, vancomycin-resistant enterococci; VV, veno-venous; XDR, extensively drug-resistant; 1-y, 1-year.

### 3.2. Infection Case Definitions

Eleven of 47 reports (23.4%) applied Tier A logic [9,10,23,28,38,39,41,52,53,57,58] and 22 (46.8%) fell into Tier B [8,11,19,20,21,27,29,31,32,33,34,35,36,37,42,46,48,49,54,55,59,60]. One (2.1%) relied on a metagenomic next-generation sequencing (mNGS) composite without a culture criterion (Tier C) [30], and 13 (27.7%) measured a different construct or did not quantify BSI [18,22,24,25,26,40,43,44,45,47,50,51,56]. Two Tier A assignments rest on explicit adoption of the NHSN LCBI criteria rather than on a restatement of the commensal rule (Joshi et al. [28]; Pieri et al. [41]); both are presented in Supplementary Table S2. Among the 27 reports with a calculable BSI proportion, the median was 19.4% in Tier A and 10.0% in Tier B, with overlapping ranges (Table 2).

### 3.3. Prevalence of Bloodstream Infection

A cohort-level BSI proportion, with a numerator of patients (or, in three reports, runs or procedures) with at least one BSI, was calculable in 27 of the 40 reports contributing to the descriptive statistics (67.5%) [9,10,11,20,23,27,29,30,31,33,34,35,37,38,39,41,44,45,46,48,49,51,52,53,54,58,59]. The median was 17.4% (IQR 8.6–24.6%; range 2.6–54.0%). The lower bound came from a predominantly cardiogenic-support cohort ascertained with CDC/NNIS criteria [61] (Hsu 2009, 3/114, 2.6%) [27], and the upper bound from a VV-ECMO cohort in which 62% of patients had COVID-19 (Uçar 2024, 27/50, 54.0%) [53]. Two cohorts that ascertained candidemia only reported 5.4% on VV-ECMO [44] and 3.8% on VA-ECMO [45]. Thirteen reports could not contribute a proportion: in four the numerator was expressed as episodes [28,36,55,57]; in one the denominator was restricted to infected patients [47]; in one only the first nosocomial infection of each patient was analyzed [8]; and in the remaining seven the study did not quantify BSI separately, counted positive cultures rather than patients, ascertained a single organism group, or did not report adult data separately [18,19,21,24,26,32,50].

### 3.4. Microbiological Profile

The included studies described Gram-negative bacilli, Gram-positive cocci, yeasts and, less often, viral reactivation. *Candida* species were the most frequently reported pathogen group, consistent with prolonged central venous access, broad-spectrum antibacterial pressure and frequent renal replacement therapy. Among bacteria, *Staphylococcus aureus* (including methicillin-resistant strains), *Klebsiella pneumoniae*, *Acinetobacter baumannii*, *Pseudomonas aeruginosa*, *Enterococcus* species (including vancomycin-resistant strains) and coagulase-negative staphylococci were each reported in several studies. *Aspergillus* species and COVID-19-associated pulmonary aspergillosis (CAPA) clustered in COVID-19-era and immunomodulated cohorts. Polymicrobial infection was reported in 32 of 47 reports (68.1%).

Microbiological data were reported in inconsistent units: some studies reported patient-level organism counts, while others reported isolates, positive cultures, infection episodes, or leading organisms only. Organism-specific prevalence could therefore not be calculated or pooled, and the findings are presented as a qualitative profile. Antimicrobial-resistance phenotypes (for example, methicillin-resistant *S. aureus*, vancomycin-resistant enterococci, extended-spectrum β-lactamase production, carbapenem resistance or a formal multidrug-resistance definition) were reported in 24 of 47 reports (51.1%) [8,9,11,18,19,21,22,23,25,27,28,31,34,35,36,39,41,47,51,53,54,57,58,59], at patient, episode or isolate level and against heterogeneous criteria, which precluded a pooled resistance prevalence.

### 3.5. Distribution of Infection Sites

BSI was the principal infectious outcome assessed. Respiratory infections, including VAP, were also commonly evaluated, followed by urinary tract infection and cannula- or catheter-related infection. Surgical-site infection, skin and soft-tissue infection, intra-abdominal infection, mediastinitis and invasive fungal disease were reported less consistently. Cannula- or catheter-site infection, the site most specific to extracorporeal support, was quantified in 7 of 47 reports (14.9%) [8,10,11,33,48,49,52]. Ortuño 2025 describes 109 patients with ECMO cannula-related infection from 12 European centers but provides no at-risk denominator [40]. Site-level frequencies are reported qualitatively for the same reason as the microbiological data.

### 3.6. Mortality and Direction of Effect

A cohort-level in-hospital mortality was reported or derivable in 27 of the 40 reports contributing to the descriptive statistics (67.5%) [9,10,11,19,20,21,23,27,28,29,30,31,32,34,35,36,37,38,39,45,46,48,49,52,54,55,58], with a median of 52.5% (IQR 45.7–62.5%; range 34.2–90.4%); three of these gave no time frame for mortality and were accepted as reported [10,29,48]. Source verification recovered cohort mortality in five studies in which it had not been extracted in the previous version (Quintana et al. [46], 37.5%; Ko et al. [34], 50.0%; Lee et al. [35], 47.4%; Nesseler et al. [39], 56.6%; Poth et al. [45], 68.6%) and corrected the end point in others: Li et al. [36] was reclassified from 28-day (43.7%) to in-hospital mortality (51.9%), and Silvetti et al. [49] from death on ECMO (27.2%) to in-hospital death among adults (71.0%).Thirteen reports could not supply a comparable value: five reported mortality only within subgroups, only for a matched sample, or not at all [18,33,44,50,59], three reported mortality only among infected patients [24,26,47], and five reported only death on ECMO or ICU mortality [8,41,51,53,57].

Because mortality metrics were not commensurable across studies, the infected-versus-uninfected comparison is also summarized by direction of effect. Of 36 reports with a mortality contrast, 27 found higher mortality among infected patients [8,9,11,20,22,24,27,29,30,31,32,34,35,36,37,38,40,42,44,45,46,49,51,54,55,57,58], and nine found no significant difference [10,23,25,39,43,48,50,52,56]. Four of the 27 were not infected-versus-uninfected comparisons within an unselected ECMO cohort: two compared groups in cohorts that also included non-ECMO patients [42,51], one compared subgroup of infected patients [40], and one compared infected patients with a propensity-matched sample [44]. Among the null results, Nesseler et al. [39] modeled healthcare-associated infection as a time-varying exposure within a multistate framework and found no association with in-hospital death (adjusted hazard ratio [HR] 1.00, 95% CI 0.79–1.26); its BSI window opened 48 h after ICU admission rather than after cannulation. In the adult stratum of the largest registry analysis, acquired fungal infection was not significantly associated with death (crude OR 0.72, 95% CI 0.49–1.06) [43]. Two reports that entered infection as a time-dependent covariate found an adjusted HR of 2.40 (95% CI 0.90–6.42) for any first nosocomial infection and death during ECMO [8] and of 1.93 (1.26–2.94) for nosocomial infection complicated by severe sepsis or septic shock and ICU death [10].

ECMO duration gave the most consistent signal: of 35 reports with the comparison, 34 (97.1%) found duration longer in infected patients [8,9,10,11,18,20,21,22,23,25,27,28,29,30,31,32,33,34,35,37,38,39,42,44,45,46,48,49,50,52,53,54,55,57]. Yang et al. [58] was the only exception, with a null rather than reversed difference (9 versus 12 days, *p* = 0.66). Kızmaz et al. [33] shows why this near unanimity is also the least interpretable finding: all 28 deaths within the first 48 h occurred in the non-infected group, and no infected patient died before infection was ascertained.

### 3.7. Reported Risk Factors and Predictors

Prolonged ECMO duration was the most frequently reported risk factor for infection (22/47, 46.8%) [9,11,18,20,21,23,25,27,28,29,30,31,32,33,35,41,43,46,48,50,53,55]. No other factor was reported by more than a quarter of the reports: illness-severity scores (9/47, 19.1%) [9,10,19,22,36,39,45,54,59], CRRT or hemodialysis (9/47, 19.1%) [21,33,35,36,45,46,48,54,58], older age (7/47, 14.9%) [24,36,43,49,50,57,59], transfusion of blood products (6/47, 12.8%) [9,21,37,38,57,58], immunosuppression including corticosteroids (6/47, 12.8%) [20,21,24,28,53,60] and invasive ventilation before ECMO (4/47, 8.5%) [18,25,29,54]. Two reports found the opposite direction for a related factor: younger age (Grasselli et al. [8]) and a shorter duration of respiratory support before ECMO (Carelli et al. [23]). Where reported, adjusted effect estimates for ECMO duration ranged from 1.06 to 1.40 per additional day of support (Hsu et al. [27], OR 1.146; Aubron et al. [9], OR 1.08; Kim et al. [32], HR 1.40; Kang et al. [30], OR 1.22; Carelli et al. [23], OR 1.06; Uçar et al. [53], OR 1.13). These estimates come from different models with different covariate sets and were not pooled. Infection itself was reported as an independent predictor of adverse outcome in 15 of 47 reports (31.9%) [8,10,20,22,24,31,36,40,44,45,46,51,55,57,59]; three assessed the association formally and found none [23,39,50], and the remaining 29 did not assess it.

### 3.8. ECMO Indication and Antimicrobial Prophylaxis

Antibacterial prophylaxis at cannulation was routine in 11 reports, restricted to selected patients or configurations in three, partially applied in one, left to clinician discretion without a protocol in six and explicitly not given in 10; 16 did not report it (Table 1). Routine antifungal prophylaxis was described in one report (fluconazole, Andersen et al.) [20]; 12 reported none, two had no protocol and 32 did not report it. Prophylaxis tracked indication. Eight of the 11 reports with routine antibacterial prophylaxis concerned cardiogenic shock, post-cardiotomy or ECPR-predominant cohorts [11,32,36,46,49,54,55,60], whereas nine of the 10 reports without prophylaxis concerned respiratory, COVID-19 or trauma populations [19,22,29,47,51,52,56,57,58] and the tenth a post-cardiotomy cohort in which only surgical prophylaxis was given [48]. The median BSI proportion was 16.9% in reports with routine antibacterial prophylaxis (six reports), 19.4% in those without it (five reports) and 20.5% where it was not protocolized (six reports). Because prophylaxis, indication and ECMO mode are aligned in this literature, these unadjusted figures cannot separate an effect of prophylaxis from case mix, and resistance could not be related to prophylaxis because it was reported in incompatible units.

### 3.9. Meta-Analysis, Outcome A: Infection and Mortality

Twenty-four studies provided complete four-cell data [8,9,10,11,20,23,27,29,30,31,32,34,35,37,38,39,45,46,48,49,54,55,57,58] (Table 3), comprising 4250 patients, runs or procedures, 1094 of them infected, and 2274 deaths. The exposure was BSI or bacteremia in 11 studies, any nosocomial infection in 12 and invasive fungal disease in one. The endpoint was in-hospital death in 13, survival to discharge in six (analyzed as its complement), ICU death in two and mortality without a stated time frame in three. Menaker et al. [37] reported mortality separately for its VV and VA subgroups, and the two strata were summarized. We reconstructed the table of Schmidt et al. [10] from reported survival percentages, and we derived the table of Selçuk et al. [48] from an adult comparison whose counts are internally inconsistent across the report. Study-level ORs ranged from 0.83 (Nesseler et al. [39]) to 56.0 (Kim et al. [31]).

**Figure 2:**
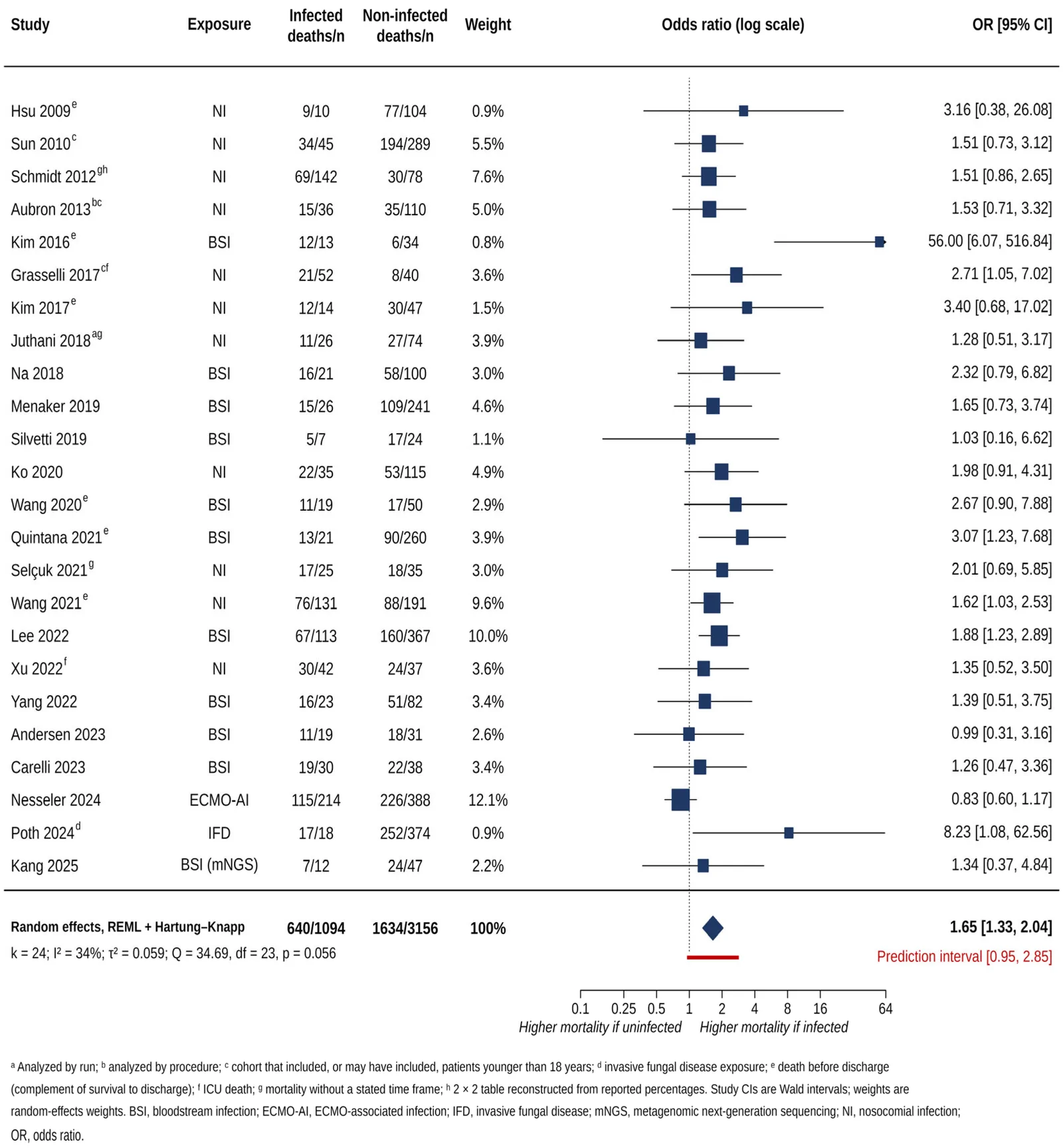
Forest plot for Outcome A: crude odds ratios for mortality in infected versus non-infected adults on ECMO in 24 studies (Hsu 2009 [27], Sun 2010 [11], Schmidt 2012 [10], Aubron 2013 [9], Kim 2016 [31], Grasselli 2017 [8], Kim 2017 [32], Juthani 2018 [29], Na 2018 [38], Menaker 2019 [37], Silvetti 2019 [49], Ko 2020 [34], Wang 2020 [54], Quintana 2021 [46], Selçuk 2021 [48], Wang 2021 [55], Lee 2022 [35], Xu 2022 [57], Yang 2022 [58], Andersen 2023 [20], Carelli 2023 [23], Nesseler 2024 [39], Poth 2024 [45], Kang 2025 [30]). The plot shows study weights, the random-effects pooled estimate (REML with Hartung–Knapp confidence interval; OR 1.65, 95% CI 1.33–2.04) and the 95% prediction interval (0.95–2.85). Footnotes: a, analyzed by run; b, analyzed by procedure; c, cohort that included, or may have included, patients younger than 18 years; d, invasive fungal disease exposure; e, death before discharge (complement of survival to discharge); f, ICU death; g, mortality without a stated time frame; h, 2 × 2 table reconstructed from reported percentages.

Infection was associated with higher crude odds of death (pooled OR 1.65, 95% CI 1.33–2.04; Figure 2). Heterogeneity was low to moderate (Q = 34.69, df = 23, *p* = 0.056; I^2^ = 34%; τ^2^ = 0.059, 95% CI 0 to 0.542) and was largely attributable to a single study (see below). The 95% prediction interval, 0.95 to 2.85, just includes the null: in a comparable future cohort, the association could plausibly range from none to an almost threefold increase in odds. Twenty-two of the 24 study-level point estimates exceeded 1.

The direction of the association held across all sensitivity analyses. Restricting the exposure to BSI or bacteremia (12 studies, with Sun et al. [11] entered with its BSI-specific counts) gave OR 1.96 (95% CI 1.39–2.77; I^2^ = 28%), and restricting the end point to in-hospital death (13 studies) gave 1.44 (1.12–1.84; I^2^ = 25%). Excluding run- or procedure-level studies gave 1.69 (1.33–2.14), excluding cohorts that included, or may have included, patients younger than 18 years gave 1.64 (1.28–2.11), and excluding the invasive fungal disease exposure gave 1.62 (1.31–2.00). Excluding Kim et al. [31], whose OR of 56 was derived from a cohort selected on the availability of catheter-tip cultures, gave 1.59 (1.34–1.89; I^2^ = 10%). Excluding the reconstructed and internally inconsistent tables (Schmidt et al. [10]; Selçuk et al. [48]) gave 1.66 (1.31–2.12), and the alternative reconstruction for Schmidt et al. [10] gave 1.65 (1.33–2.05). Adding the adult crude OR reported by Pluim et al. [43], which gives no cell counts, gave 1.57 (1.25–1.98; I^2^ = 49%; 25 studies), and adding Poth et al. [44], whose comparator was a propensity-matched sample, gave 1.70 (1.36–2.13; 25 studies). Because Wang et al. [55] and Li et al. [36] overlap, only Wang et al. [55] was included in Outcome A. We reproduced the 20-study estimate from the previous version (1.65, 1.27–2.14) before adding the reinstated reports.

Leave-one-out estimates ranged from 1.59 to 1.78. Nesseler et al. [39] was the only influential study (studentized residual −4.18; Cook’s distance 0.54); without it the OR rose to 1.78 (1.49–2.12) and τ^2^ fell to zero. This reflects removal of the largest cohort, whose crude estimate was null, rather than genuine homogeneity among the others. Kim et al. [31] was an outlier (studentized residual 3.75) but carried 0.8% of the weight and was not influential. In exploratory subgroup analyses excluding the invasive fungal disease exposure, the OR was 1.88 (1.31–2.68; 11 studies) when the exposure was BSI and 1.48 (1.17–1.87; 12 studies) when it was any nosocomial infection (*p* = 0.22 for the difference). By indication, it was 1.10 (0.76–1.59) in respiratory cohorts including COVID-19 (six studies), 1.81 (1.41–2.33) in cardiac cohorts (nine studies) and 1.89 (1.29–2.76) in mixed cohorts (nine studies) (F(2,21) = 6.57, *p* = 0.006). By COVID-19 cohort status (post hoc), it was 0.88 (0.60–1.29) in the three COVID-19 cohorts and 1.83 (1.52–2.19) in the 21 pre-pandemic cohorts (F(1,22) = 19.41, *p* < 0.001). Indication, ECMO mode and pandemic era are collinear, because the respiratory, VV-predominant and COVID-19 subgroups share their studies, and these contrasts are hypothesis-generating.

Egger’s regression suggested small-study effects (bias coefficient 1.53, t = 3.43, df = 22, *p* = 0.002), as did the REML-based Egger-type test (*p* = 0.004), whereas Begg’s rank correlation (*p* = 0.29) and Peters’ test (*p* = 0.21) did not. The Egger signal weakened when Nesseler et al. [39] was omitted (*p* = 0.062) but persisted when Kim et al. [31] was omitted (*p* = 0.009). Trim-and-fill imputed nine studies with the L0 estimator (adjusted OR 1.38, 1.08–1.76) and six with the R0 estimator (1.46, 1.13–1.87) (Supplementary Figure S1). The registered DerSimonian–Laird model gave 1.67 (1.35–2.07) and the Mantel–Haenszel common-effect model 1.56 (1.34–1.82); REML with Wald intervals gave 1.65 (1.35–2.02), Paule–Mandel with Hartung–Knapp intervals 1.65 (1.33–2.05) and the binomial–normal GLMM 1.70 (1.39–2.09).

### 3.10. Meta-Analysis, Outcome B: Pooled BSI Prevalence

Seven studies met all three conditions: Schmidt 2012 (39/220) [10], Pieri et al. (3/27) [41], Na et al. (21/121) [38], Yang et al. (23/105) [58], Carelli et al. (30/68) [23], Nesseler et al. (154/602) [39] and Uçar et al. (27/50) [53], comprising 297 BSI events among 1193 patients (Table 4). Individual prevalences ranged from 11.1% to 54.0%. Four other Tier A reports were ineligible: Aubron 2013 [9] and Thomas 2017 [52] counted procedures or runs, and Joshi 2024 [28] and Xu 2022 [57] counted episodes. Sun 2010 [11], reclassified to Tier B on re-audit, contributes only to a sensitivity analysis. Four cohorts were VV only, one was VV-predominant, one was VA only and one was mixed; overall, 76% of patients were on VV-ECMO, and 60% came from the three cohorts in which at least half of the patients had COVID-19.

**Figure 3:**
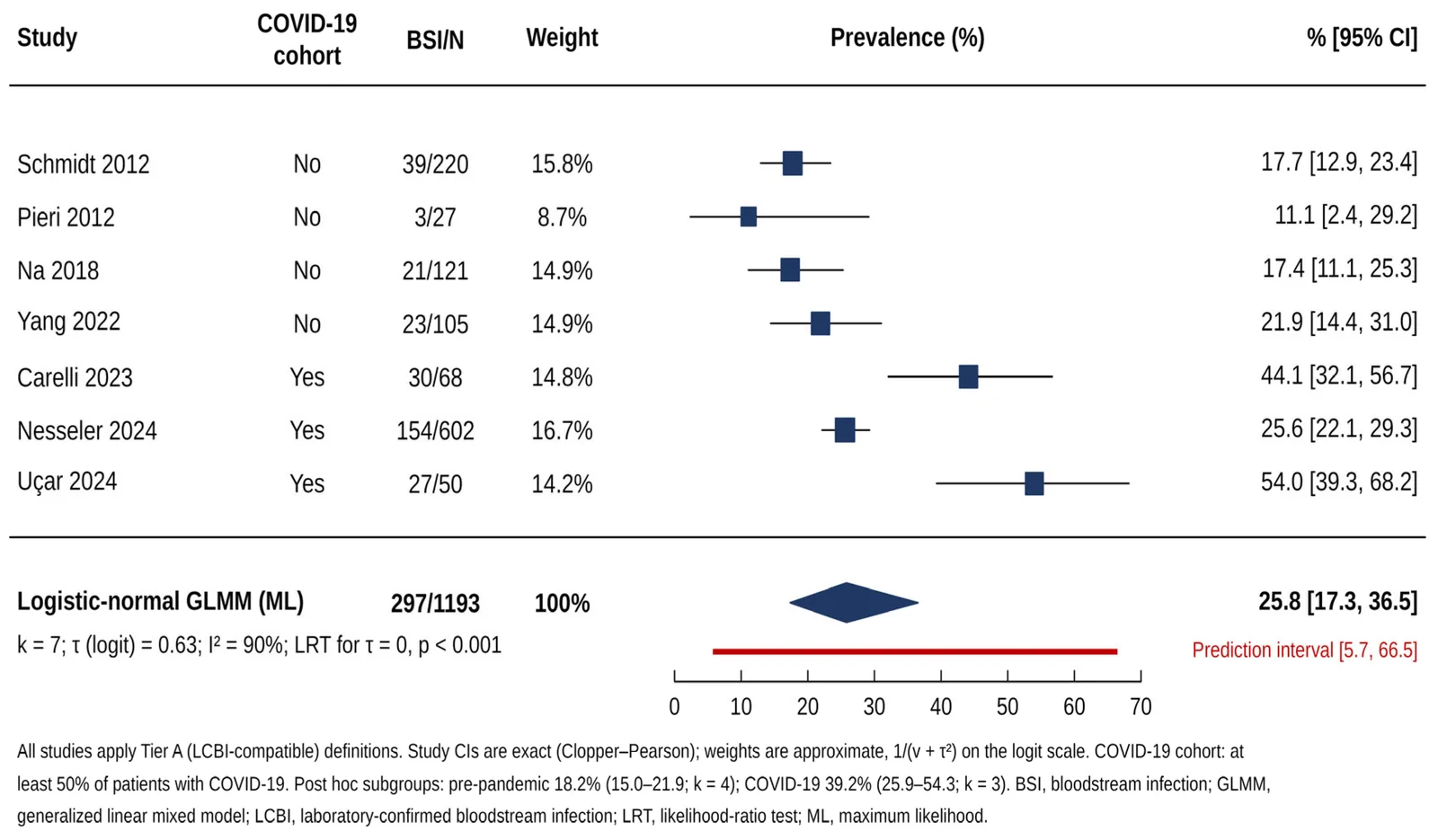
Forest plot for Outcome B: patient-level bloodstream infection prevalence in the seven Tier A studies (Schmidt 2012 [10], Pieri 2012 [41], Na 2018 [38], Yang 2022 [58], Carelli 2023 [23], Nesseler 2024 [39], Uçar 2024 [53]). The plot shows exact (Clopper–Pearson) study-level confidence intervals, approximate study weights and the pooled logistic-normal generalized linear mixed model estimate (25.8%, 95% CI 17.3–36.5%; 95% prediction interval 5.7–66.5%).

The GLMM gave a pooled patient-level BSI prevalence of 25.8% (95% CI 17.3–36.5%), with a logit-scale τ of 0.63 (I^2^ = 90.3%; likelihood-ratio test for τ = 0, *p* < 0.001) and a 95% prediction interval of 5.7–66.5% (Figure 3). The profile-likelihood CI was 15.9–38.3%. Approximate study weights ranged from 8.7% (Pieri et al. [41]) to 16.7% (Nesseler et al. [39]). Excluding Pieri et al. [41] gave 28.0% (18.9–39.2%; six studies); substituting the later report of the same cohort (Pieri et al. [42], 8/46) gave 26.5% (18.4–36.4%); and adding Sun et al. [11] (34/334) gave 22.9% (15.0–33.5%; τ = 0.71). The registered Freeman–Tukey model with DerSimonian–Laird estimation gave 26.2% (18.7–34.5%). Heterogeneity was partly explained by pandemic era (post hoc): prevalence was 18.2% (15.0–21.9%; τ = 0) in the four pre-pandemic cohorts and 39.2% (25.9–54.3%) in the three COVID-19 cohorts (meta-regression OR 2.86, 95% CI 1.51–5.43; *p* = 0.001; residual τ 0.34). The estimate applies to infections meeting LCBI-compatible criteria in similar cohorts; episode-based rates, isolate counts, and infections per 1000 ECMO-days have different denominators and were not combined. It exceeds the unweighted descriptive median of 17.4% across the 27 reports with a cohort-level proportion, whereas the pre-pandemic pooled estimate is close to that median.

### 3.11. Risk of Bias

**Figure 4:**
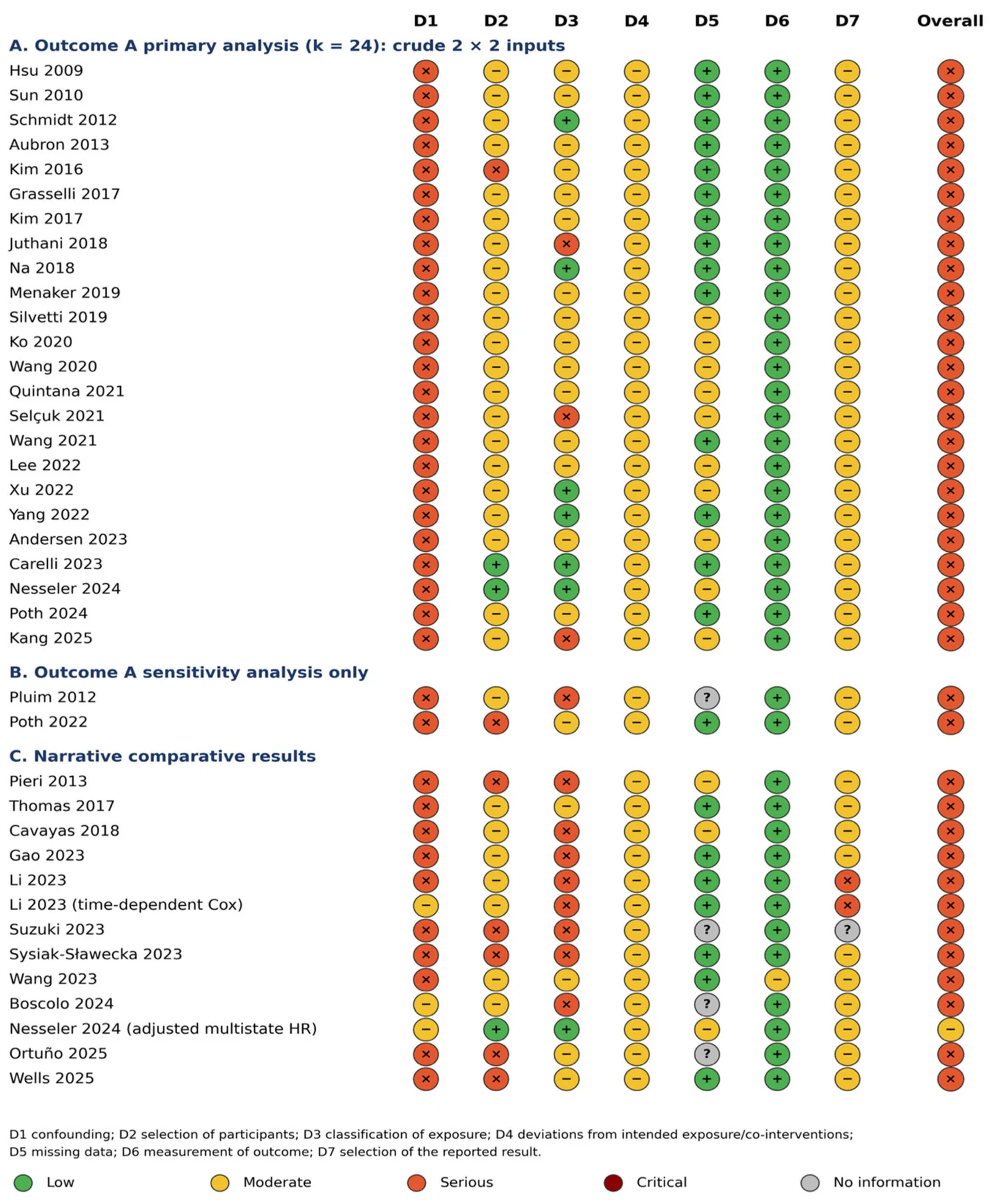
ROBINS-I [62] assessment, by domain and overall, of three groups of results: (**A**) The 24 results in the primary mortality meta-analysis (Outcome A): Hsu 2009 [27], Sun 2010 [11], Schmidt 2012 [10], Aubron 2013 [9], Kim 2016 [31], Grasselli 2017 [8], Kim 2017 [32], Juthani 2018 [29], Na 2018 [38], Menaker 2019 [37], Silvetti 2019 [49], Ko 2020 [34], Wang 2020 [54], Quintana 2021 [46], Selçuk 2021 [48], Wang 2021 [55], Lee 2022 [35], Xu 2022 [57], Yang 2022 [58], Andersen 2023 [20], Carelli 2023 [23], Nesseler 2024 [39], Poth 2024 [45] and Kang 2025 [30]. (**B**) The two results entering sensitivity analyses only: Pluim 2012 [43] and Poth 2022 [44]. (**C**) The comparative results reported narratively: Pieri 2013 [42], Thomas 2017 [52], Cavayas 2018 [24], Gao 2023 [25], Li 2023 [36], including its time-dependent Cox estimate, Suzuki 2023 [50], Sysiak-Sławecka 2023 [51], Wang 2023 [18], Boscolo 2024 [22], Nesseler 2024 [39], including its adjusted multistate estimate, Ortuño 2025 [40] and Wells 2025 [56].

All 37 comparative results assessed with ROBINS-I were at serious overall risk of bias, and none was at critical risk (Figure 4). Confounding drove the judgment: each result compared ever-infected with never-infected patients, using crude or incompletely adjusted estimates that did not account for the time-dependent nature of infection. The crude 2 × 2 result from Nesseler et al. [39] used in Outcome A was also at serious risk; its adjusted multistate estimate, reported narratively, was at moderate risk, because the model addressed time dependence although residual confounding could not be excluded. In the 24 Outcome A results, classification of the exposure was at low risk in six, moderate in 15 and serious in three, tracking the operational strength of each case definition; selection of participants was at serious risk in one (Kim et al. [31], enrollment conditional on catheter-tip culture availability), and two results carry additional uncertainty from reconstructed or internally inconsistent counts (Schmidt et al. [10]; Selçuk et al. [48]). Outcome measurement was at low risk throughout, death being an objective endpoint.

With the JBI prevalence checklist, 13 of 47 reports were of low concern, 16 of moderate concern and 18 of high concern (Supplementary Figure S2). Of the seven Outcome B studies, five were of low concern (Schmidt et al. [10], Na et al. [38], Yang et al. [58], Carelli et al. [23] and Uçar et al. [53]), Nesseler et al. [39] was of moderate concern and Pieri et al. [41] of high concern (case–control sampling and a BSI numerator not confirmed as wholly ECMO-acquired). Studies of moderate or high concern carried 25% of the pooled weight. In six of the seven, cultures were obtained on clinical suspicion rather than by surveillance.

### 3.12. Certainty of Evidence

Both outcomes started at high certainty [65,66] and ended at very low certainty (Table 5). For the mortality association, we rated down two levels for risk of bias, because every result was at serious risk from confounding and immortal time, and one level for publication bias, given the Egger signal and the attenuation to OR 1.38 with trim-and-fill. We counted heterogeneity of case definitions once under risk of bias. Inconsistency (I^2^ = 34%, largely attributable to a single study), indirectness and imprecision were not judged serious. Against a median mortality of 512 per 1000 among uninfected patients, the pooled OR corresponds to 634 per 1000 among infected patients (95% CI 583–682), or 121 more deaths per 1000 (70 to 170 more). For BSI prevalence, we rated down one level each for risk of bias (cultures obtained on clinical suspicion in six of seven studies, which may under-ascertain BSI, and a BSI window anchored to ICU admission in the largest cohort, which may over-count it), inconsistency (prediction interval 5.7–66.5%, partly explained by the pandemic era) and imprecision (95% CI 17.3–36.5%). The anticipated prevalence is 258 per 1000 (173 to 365). We are therefore uncertain whether infection acquired during ECMO increases mortality, and uncertain how closely the pooled prevalence applies to any particular ECMO population.

## 4. Discussion

This review synthesizes 47 reports describing 46 cohorts of adults on ECMO and quantifies both the burden and the prognostic association of nosocomial and bloodstream infection acquired during support. In the seven studies that used an LCBI-consistent definition with a recoverable patient-level numerator and denominator, pooled BSI prevalence was 25.8% (95% CI 17.3–36.5%), with wide variation partly explained by the pandemic era (18.2% in pre-pandemic and 39.2% in COVID-19 cohorts). In the 24 studies with a reconstructable 2 × 2 mortality table, the crude OR for death in infected versus uninfected patients was 1.65 (95% CI 1.33–2.04; I^2^ = 34%), in the same direction as primary cohorts [8,10]. Median study-level in-hospital mortality was 52.5% (IQR 45.7–62.5%), in keeping with the severity of illness in this population. Gram-negative bacilli, particularly *K. pneumoniae*, *A. baumannii* and *P. aeruginosa*, together with *Candida* species, dominated the microbiological profile.

The behavior of the mortality estimate across analytic choices is more informative than its point value. Restricting the exposure to BSI strengthened the association (OR 1.96), restricting the endpoint to in-hospital death attenuated it slightly (OR 1.44), and correction for small-study effects reduced it to 1.38 (95% CI 1.08–1.76). The prediction interval (0.95 to 2.85) includes no association. The pooled OR describes how much more often infected patients died in these cohorts; it does not estimate how many deaths infection causes.

Most contributing studies treated infection as a fixed ever-versus-never exposure rather than a time-dependent covariate, which biases the comparison toward higher mortality in the infected group. Patients who die within the first 48 to 72 h of cannulation have no opportunity to acquire a nosocomial infection. One cohort illustrates the mechanism: all 28 deaths in the first 48 h occurred in the non-infected group, and no infected patient died before infection was ascertained [33]. Analyses that account for time give smaller or less certain estimates. In Li et al., a competing-risk model adjusted only for age, sex and SOFA score gave an HR of 1.50, whereas a time-dependent Cox model gave 1.05 (95% CI 1.00–1.11); that study’s exposure also included infections arising after decannulation [36]. Nesseler et al., which modeled healthcare-associated infection as a time-varying state within a multistate model, found no association with in-hospital death (adjusted HR 1.00, 95% CI 0.79–1.26), although its infection window was anchored to ICU admission rather than cannulation [39]. The same cohort’s crude 2 × 2 table gave the lowest point estimate in Outcome A (OR 0.83) and was the single influential study; omitting it raised the pooled OR to 1.78 and removed the heterogeneity. In Grasselli et al., a first nosocomial infection entered as a time-dependent covariate gave an adjusted HR of 2.40 for death during ECMO, with a CI that included the null (0.90–6.42) [8]; in Schmidt et al., only infection complicated by severe sepsis or septic shock was modeled in this way, and it was associated with ICU death (adjusted HR 1.93, 1.26–2.94) [10]. The pooled OR is best read as a crude association that most plausibly overestimates any causal effect, not as a causal parameter.

By ECMO mode, median in-hospital mortality was similar in VA-predominant (55.1%; IQR 48.8–68.7%; 12 reports) and VV-predominant cohorts (58.0%; IQR 56.3–61.2%; 9 reports), with overlapping ranges. BSI proportions separated more clearly: a median of 25.6% (IQR 19.4–40.0%; 9 reports) in VV-predominant cohorts against 9.9% (IQR 4.8–16.7%; 10 reports) in VA-predominant cohorts. The difference is real in the descriptive data but cannot be attributed to configuration, because mode and case mix are confounded by design in this literature. All six COVID-19-era cohorts, the partly pandemic-era cohort and a cohort in which 62% of patients had COVID-19 fall within the VV group [20,21,22,23,28,39,51,53], and pooled prevalence was more than twice as high in COVID-19 as in pre-pandemic cohorts. Those patients were supported for longer, were more often treated with corticosteroids and interleukin-6-receptor antagonists, and were more intensively surveilled. VA-predominant cohorts were dominated by cardiogenic shock and post-cardiotomy populations with shorter runs. A risk-factor meta-analysis identified VA-ECMO as a correlate of nosocomial infection [69], but without individual patient data and rigorous adjustment, any mode-attributable inference is premature.

Indication showed a pattern opposite to prevalence. In the exploratory Outcome A subgroups, the crude OR was 1.81 in cardiac cohorts and 1.10 in respiratory cohorts, and 0.88 in the three COVID-19 cohorts, compared with 1.83 in pre-pandemic cohorts. The respiratory and COVID-19 subgroups include Nesseler et al. [39], the largest study and the one with the lowest crude estimate, and indication is collinear with mode and pandemic era, so the contrast may reflect case mix, baseline mortality from the underlying lung disease, or the analytic approach rather than a different prognostic weight of infection. It is best treated as a hypothesis for studies that stratify by indication from the outset.

Antimicrobial prophylaxis could not be evaluated as an exposure. Routine antibacterial prophylaxis was described in 11 reports, eight in cardiogenic shock, post-cardiotomy, or ECPR cohorts, and nine of the 10 reports without prophylaxis concerned respiratory, COVID-19, or trauma populations; median BSI proportions were similar across prophylaxis categories. Routine antifungal prophylaxis appeared in a single report, despite Candida’s prominence in the microbiology. The only pooled evidence, a meta-analysis of 7996 patients, found no 30-day mortality benefit from antibiotic prophylaxis and a marginal reduction in nosocomial infection, at very low certainty [70], and ELSO has not endorsed a standard prophylaxis policy [1]. Prophylaxis protocols should be among the minimum reporting elements for future ECMO infection studies [15], so their effect on BSI and resistance can be separated from indication.

A high frequency of infection is biologically plausible. Continuous exposure of blood to the non-endothelialized circuit activates the contact system, indiscriminately primes innate immunity, and raises circulating tumor necrosis factor-α, interleukin-6, and interleukin-8, producing hyperinflammation and functional immune-paresis at the same time [7]. Hypoperfusion, oxidative stress and vasoactive drugs disrupt the intestinal epithelial barrier and may promote translocation of multidrug-resistant Gram-negative bacteria, one explanation for their dominance here and in earlier series [8,25]. Large-bore cannulas, endotracheal tubes, central venous catheters, and urinary catheters add cumulative mechanical breaches that, across 25 reports providing cohort-level estimates, lasted a median of 9.8 days (IQR 7.7–14.0). Prolonged support was the most consistently reported correlate of infection, with a dose–response relationship in several cohorts [29,60]. A risk-factor meta-analysis identified immunosuppression, heart transplantation, VA-ECMO, CRRT, red-cell transfusion, duration of ventilation and length of ICU and hospital stay as pooled correlates [69]. These map onto three intersecting mechanisms: an immunocompromised host, device-related breach of anatomical barriers, and the selection pressure of prolonged broad-spectrum antimicrobial exposure. The duration association also resists causal reading, because prolonged support both allows infection to be acquired and results from infection-related failure to wean.

Pharmacokinetic derangement imposed by the circuit is an under-recognized contributor to treatment failure and resistance. Lipophilic and highly protein-bound antimicrobials can be sequestered by oxygenator and tubing polymers, and volumes of distribution expand, so free-drug exposure at the site of infection may fall below target [71]. The effect is drug-specific rather than universal, which argues for therapeutic drug monitoring and model-informed dosing, particularly with concomitant CRRT [71]. Resistance phenotypes were described in 24 of 47 reports, but mostly as named resistant organisms rather than as proportions of isolates meeting a formal definition. That is an evidence gap, not reassurance, and it hides the resistance cost of contemporary ECMO practice.

The COVID-19-era VV cohorts formed a distinct group [20,21,23,28,39,51]. Prolonged ventilation and support coincided with intensive immunomodulation, conditions that favor bacterial superinfection, CAPA, reactivation of cytomegalovirus and herpes simplex virus, and invasive candidiasis. Immunomodulation in patients on ECMO therefore must be weighed against the fungal and viral reactivation risk it carries, and pre-emptive galactomannan, β-D-glucan and molecular surveillance strategies warrant prospective evaluation.

These findings support embedding infectious-disease expertise within adult ECMO programs rather than relying on consultation on request. The framework proposed by Marcus and colleagues, comprising diagnostic stewardship, peri-cannulation prophylaxis calibrated to the local antibiogram, standardized case definitions and pharmacist-led dosing, offers a practical template [1]. Diagnostic stewardship matters most: reflexive pan-culturing of a persistently inflamed, febrile patient on ECMO risks treating colonization as infection, with unnecessary broad-spectrum exposure, *Clostridioides difficile* colitis and further selection of resistance [1]. A recent review of stewardship during ECMO reached a similar conclusion: interprofessional programs that combine judicious antimicrobial use with non-pharmacologic prevention, such as strict asepsis and adherence to preventive bundles, may reduce antimicrobial consumption and costs without compromising outcomes, although ECMO-specific evidence is limited and much of current practice is extrapolated from general intensive care [16]. No adequately powered randomized trial has evaluated prophylaxis, surveillance or infection-directed intervention in adults on ECMO. Pragmatic cluster-randomized designs embedded in registry infrastructure and stratified by mode and indication offer a plausible way forward.

The consequences extend beyond the individual patient. Infected patients had longer ECMO runs in 34 of 35 reports with the comparison, with corresponding demands on circuits, beds and staff [8]. At the ecological level, the dominance of multidrug-resistant Gram-negative pathogens, together with prolonged carbapenem, polymyxin and glycopeptide exposure, makes the ECMO population a potential amplifier of hospital resistance. Individual cohorts reported carbapenem resistance in 90% of *A. baumannii* isolates and vancomycin resistance in 58% of enterococci [59], multidrug resistance in 58% of isolates [53], and the emergence of extensively or pan-resistant Gram-negative organisms during prolonged runs [21,25,57].

The review covers the literature to December 2025 and includes 47 reports. It separates a narrative layer from a targeted meta-analytic layer so it describes definitional heterogeneity rather than averaging it away. Classifying case definitions by the criteria applied rather than by citation changed the classification of five of the seven definitions first audited and of one further study at re-audit. During revision, we re-verified all values used in tables and pooled analyses against the primary publications; re-examined every full-text exclusion; declared deviations from the registered protocol; and reported estimates from the registered methods alongside the final ones. The review also makes the immortal-time problem explicit and identifies specific gaps: harmonized definitions, systematic resistance reporting, time-dependent modeling of the infection–mortality relationship, prospective evaluation of stewardship interventions and pharmacokinetically guided dosing trials.

The limitations are substantial. The evidence is exclusively observational, and every comparative result was at serious risk of bias, chiefly from confounding and time-dependent exposure. The review was not prospectively registered, and several methodological choices, including the tier framework, the change in pooling model, the widened Outcome A set and the COVID-19 subgroup analyses, were made after registration; they are declared in Section 2.1, and the registered methods gave similar estimates. We excluded six eligible reports at the original full-text screen and reinstated them only on re-audit. Only 11 of 47 reports implemented an LCBI definition, and few applied a formal resistance definition to all isolates. The pooled prevalence rests on seven heterogeneous studies, 60% of whose patients came from COVID-19 cohorts; its Wald interval is conditional on the estimated between-study variance, as the wider prediction interval shows. Egger’s test suggested small-study effects in the mortality analysis. Five cohorts included, or may have included, patients younger than 18 years; two pairs of reports have overlapping populations (Pieri et al. [41,42]; Li et al. [36] and Wang et al. [55]); two Outcome A tables were reconstructed from percentages or were internally inconsistent (Schmidt et al. [10]; Selçuk et al. [48]); one ELSO registry analysis was published as a case report and counted Aspergillus isolation that may include colonization [26]; and one cohort included patients on pumpless extracorporeal lung assist [19]. Four reports analyzed runs or procedures rather than patients, and four expressed BSI numerators as episodes, so none of them could contribute a patient-level prevalence. No included study reported outcomes at 30 days, 90 days, 6 months or 12 months after decannulation. Microbiology was reported in inconsistent units, so we could not pool organism-specific prevalence. The English-language restriction and the dominance of European, North American and East Asian cohorts limit generalizability: ECMO programs in Latin America, Africa and South Asia are under-represented, and their pathogen and resistance ecologies remain uncharacterized.

Priorities for the field are harmonized case definitions [14,15], time-dependent analysis of outcomes, systematic reporting of resistance and prophylaxis [70], embedded infectious-disease and intensive-care collaboration with diagnostic and antimicrobial stewardship [16], pharmacokinetically guided dosing [71], and pragmatic randomized designs to test candidate interventions.

## 5. Conclusions

Bloodstream and respiratory-tract infections are common during adult ECMO, and Gram-negative bacilli and *Candida* species predominate. In cohorts using laboratory-confirmed criteria, about one in four patients (25.8%) developed BSI, with wide variation partly explained by the pandemic era (18.2% before and 39.2% during the COVID-19 pandemic). Infected patients had higher crude odds of death (OR 1.65), but the prediction interval included no effect; small-study effects were suggested, and analyses that modeled infection as a time-varying exposure gave inconsistent results, including no association in the largest cohort. With very low certainty for both outcomes, we are uncertain whether infection acquired during ECMO increases mortality.

## Figures and Tables

**Table 2:** Cohort-level BSI proportion by case-definition tier (27 reports).

Definition Tier	Reports	Median (%)	IQR (%)	Range (%)
Tier A (LCBI logic)	9	19.4	17.4–25.6	11.1–54.0
Tier B (no commensal rule)	14	10.0	7.2–23.3	2.6–38.0
Tier C (mNGS composite)	1	20.3	NA	NA
Not assigned	3	5.4	4.6–22.7	3.8–40.0
All	27	17.4	8.6–24.6	2.6–54.0

Unweighted distribution of study-level proportions; not pooled estimates. Quartiles by linear interpolation. Numerators are patients with at least one BSI, except Aubron et al. [9] (procedures) and Juthani et al. [29] and Thomas et al. [52] (runs). Tier A: Schmidt et al. [10], Aubron et al. [9], Pieri et al. [41], Thomas et al. [52], Na et al. [38], Yang et al. [58], Carelli et al. [23], Nesseler et al. [39] and Uçar et al. [53]. Not assigned (tier NA in Table 1): Poth et al. [44,45] (candidemia only) and Sysiak-Sławecka et al. [51] (positive blood cultures at any time during the ICU stay). Not included: Joshi et al. [28] and Xu et al. [57] (Tier A) and Li et al. [36] and Wang et al. [55] (Tier B), all with episode-based numerators; Pieri et al. [42], the same cohort as Pieri et al. [41]. NA, not applicable (single report). BSI, bloodstream infection; IQR, interquartile range; LCBI, laboratory-confirmed bloodstream infection; mNGS, metagenomic next-generation sequencing.

**Table 3:** Outcome A: study-level data and crude effect estimates (24 studies).

Study	Exposure	End Point	Infected: Deaths/n	Non-Infected: Deaths/n	Crude OR (95% CI)
Hsu 2009 [27]	Any NI	Survival to discharge ^c^	9/10	77/104	3.16 (0.38–26.08)
Sun 2010 [11] ^b^	Any NI	In-hospital	34/45	194/289	1.51 (0.73–3.12)
Schmidt 2012 [10] ^f^	Any NI	Mortality as reported	69/142	30/78	1.51 (0.86–2.65)
Aubron 2013 [9] ^ab^	Any NI (procedures)	In-hospital	15/36	35/110	1.53 (0.71–3.32)
Kim 2016 [31] ^e^	BSI	Survival to discharge ^c^	12/13	6/34	56.00 (6.07–516.84)
Grasselli 2017 [8] ^b^	Any NI	ICU death	21/52	8/40	2.71 (1.05–7.02)
Kim 2017 [32]	Any NI	Survival to discharge ^c^	12/14	30/47	3.40 (0.68–17.02)
Juthani 2018 [29] ^a^	Any NI (runs)	Mortality as reported	11/26	27/74	1.28 (0.51–3.17)
Na 2018 [38]	BSI	In-hospital	16/21	58/100	2.32 (0.79–6.82)
Menaker 2019 [37]	BSI	In-hospital ^d^	15/26	109/241	1.65 (0.73–3.74)
Silvetti 2019 [49]	BSI	In-hospital (adults)	5/7	17/24	1.03 (0.16–6.62)
Ko 2020 [34]	Any NI	In-hospital	22/35	53/115	1.98 (0.91–4.31)
Wang 2020 [54]	BSI	Survival to discharge ^c^	11/19	17/50	2.67 (0.90–7.88)
Quintana 2021 [46] ^h^	BSI	Survival to discharge ^c^	13/21	90/260	3.07 (1.23–7.68)
Selçuk 2021 [48] ^g^	Culture-proven NI (adults)	Mortality as reported	17/25	18/35	2.01 (0.69–5.85)
Wang 2021 [55]	Any NI	Survival to discharge ^c^	76/131	88/191	1.62 (1.03–2.53)
Lee 2022 [35]	BSI	In-hospital	67/113	160/367	1.88 (1.23–2.89)
Xu 2022 [57]	Any NI	ICU death	30/42	24/37	1.35 (0.52–3.50)
Yang 2022 [58]	BSI	In-hospital	16/23	51/82	1.39 (0.51–3.75)
Andersen 2023 [20]	BSI	In-hospital	11/19	18/31	0.99 (0.31–3.16)
Carelli 2023 [23]	BSI	In-hospital	19/30	22/38	1.26 (0.47–3.36)
Nesseler 2024 [39]	Any NI	In-hospital	115/214	226/388	0.83 (0.60–1.17)
Poth 2024 [45]	Invasive fungal disease	In-hospital	17/18	252/374	8.23 (1.08–62.56)
Kang 2025 [30]	BSI (mNGS)	In-hospital	7/12	24/47	1.34 (0.37–4.84)
**Pooled (REML + Hartung–Knapp)**			**640/1094**	**1634/3156**	**1.65 (1.33–2.04)**

^a^ Analyzed by ECMO run or procedure. ^b^ Cohort that included, or may have included, patients younger than 18 years. ^c^ Survival to discharge analyzed as its complement, in-hospital death. ^d^ Sum of the VV and VA strata reported by the authors; one VV patient without outcome data (267 of 268 patients). ^e^ Enrollment restricted to 47 of 72 eligible cases with catheter-tip cultures; excluded in a sensitivity analysis (OR 1.59, 1.34–1.89). ^f^ Reconstructed from reported survival percentages (uninfected 62% of 78; infected 51% of 142, with 73 survivors chosen because it reproduces the reported overall survival of 55%); the alternative reconstruction (70/142 deaths) gives a study OR of 1.56 and a pooled OR of 1.65 (1.33–2.05). ^g^ Adult culture-positive vs. culture-negative counts of infected adults differ across the report. ^h^ VA and VV strata only (281 of 288 patients); the seven VAV patients were not reported by infection status. Crude ORs are unadjusted and calculated from the reported counts; study-level CIs are Wald intervals. In the BSI-only sensitivity analysis, Sun et al. [11] was entered with its BSI-specific counts (30/34 vs. 198/300). BSI, bloodstream infection; CI, confidence interval; ECMO, extracorporeal membrane oxygenation; ICU, intensive care unit; mNGS, metagenomic next-generation sequencing; NI, nosocomial infection; OR, odds ratio; REML, restricted maximum likelihood; VA, veno-arterial; VAV, veno-arteriovenous; VV, veno-venous.

**Table 4:** Outcome B: studies in the pooled patient-level BSI prevalence analysis, with the logistic-normal generalized linear mixed model estimate.

Study	ECMO Mode	COVID-19 Cohort	BSI/Patients	Prevalence, % (95% CI)	Weight (%)
Schmidt 2012 [10]	VA only	No	39/220	17.7 (12.9–23.4)	15.8
Pieri 2012 [41]	Mixed (VA 59%, VV 41%)	No	3/27	11.1 (2.4–29.2)	8.7
Na 2018 [38]	VV only	No	21/121	17.4 (11.1–25.3)	14.9
Yang 2022 [58]	VV only	No	23/105	21.9 (14.4–31.0)	14.9
Carelli 2023 [23]	VV only	Yes	30/68	44.1 (32.1–56.7)	14.8
Nesseler 2024 [39]	VV-predominant (VV 91%)	Yes	154/602	25.6 (22.1–29.3)	16.7
Uçar 2024 [53]	VV only	Yes ^a^	27/50	54.0 (39.3–68.2)	14.2
Pooled (GLMM)			297/1193	25.8 (17.3–36.5)	100
Sun 2010 [11] ^b^	VA-predominant	No	34/334	10.2 (7.2–13.9)	NA
Pieri 2013 [42] ^c^	Mixed (ECMO subset)	No	8/46	17.4 (7.8–31.4)	NA

All seven studies apply Tier A (LCBI-compatible) definitions. Study-level CIs are exact (Clopper–Pearson). Weights are approximate, 1/(v + τ^2^) on the logit scale. Between-study τ (logit) 0.63; I^2^ 90.3%; 95% prediction interval 5.7–66.5%. Post hoc subgroups: pre-pandemic cohorts 18.2% (15.0–21.9%; 4 studies; τ = 0); COVID-19 cohorts 39.2% (25.9–54.3%; 3 studies). ^a^ COVID-19 in 62% of patients (cohorts with at least 50% of patients with COVID-19 were classed as COVID-19 cohorts). ^b^ Sensitivity analysis only (Tier B after operational re-audit); numerator = patients with at least one BSI (34), derived from the reported case fatality among patients with BSI (30/34); the 38 BSI reported elsewhere in the source are episodes. Adding Sun 2010 gives 22.9% (15.0–33.5%). ^c^ Sensitivity analysis only: later report of the Pieri 2012 cohort (Tier B), replacing Pieri 2012; gives 26.5% (18.4–36.4%). NA, not applicable (not in the primary model). BSI, bloodstream infection; GLMM, generalized linear mixed model; LCBI, laboratory-confirmed bloodstream infection; VA, veno-arterial; VV, veno-venous.

**Table 5:** GRADE evidence profile for the two pooled outcomes. Both outcomes started at high certainty (Outcome A as ROBINS-I-assessed evidence; Outcome B as event rates from observational cohorts).

Outcome	Studies and Participants	Risk of Bias	Inconsistency	Indirectness	Imprecision	Publication Bias	Effect (95% CI)	Certainty *
Mortality, infected vs. uninfected patients (Outcome A)	24 observational; 2274 deaths in 4250 patients, runs or procedures	Very serious (−2) ^a^	Not serious ^b^	Not serious ^c^	Not serious ^d^	Strongly suspected (−1) ^e^	OR 1.65 (1.33–2.04); PI 0.95–2.85. 512 per 1000 without infection vs. 634 per 1000 with infection: 121 more (70 to 170 more) ^f^	⊕◯◯◯ Very low
Patient-level BSI prevalence, Tier A definitions (Outcome B)	7 observational; 297 patients with BSI among 1193	Serious (−1) ^g^	Serious (−1) ^h^	Not serious ^i^	Serious (−1) ^j^	Not assessable ^k^	25.8% (17.3–36.5); PI 5.7–66.5. 258 per 1000 (173 to 365)	⊕◯◯◯ Very low

* ⊕⊕⊕⊕, high certainty: we are very confident that the true effect lies close to the estimate. ⊕⊕⊕◯, moderate certainty: the true effect is likely to be close to the estimate but may be substantially different. ⊕⊕◯◯, low certainty: the true effect may be substantially different from the estimate. ⊕◯◯◯, very low certainty: the true effect is likely to be substantially different from the estimate. (−1), rated down one level; (−2), rated down two levels. Superscript letters (^a–k^) refer to the explanatory notes below the table. Certainty started high for both outcomes [65,66]; each limitation was counted in one domain only. ^a^ All 24 contrasts at serious ROBINS-I risk from confounding and immortal time; exposure classification (domain 3): six low, 15 moderate, three serious. ^b^ I^2^ = 34%; excluding the single influential study (Nesseler et al. [39]) gives OR 1.78 (1.49–2.12) with τ^2^ = 0. ^c^ Restriction to in-hospital death, patient-level units or age-compliant cohorts did not change the estimate. ᵈ The CI excludes the null; 2274 events. ^e^ Egger *p* = 0.002; trim-and-fill OR 1.38 (1.08–1.76). ^f^ Assumed risk is the median mortality in uninfected patients; the difference reflects an association, not attributable deaths. ^g^ JBI: five reports at low, one at moderate and one at high concern; cultures were clinically triggered in six of seven reports. ^h^ I^2^ = 90.3%, partly explained by COVID-19 status (post hoc): 18.2% in pre-COVID-19 cohorts (k = 4) vs. 39.2% in COVID-19 cohorts (k = 3). ^i^ Mostly VV and COVID-19 cohorts; COVID-19 is counted under inconsistency only. ^j^ The CI spans about one in six to more than one in three patients. ^k^ Too few studies for small-study tests. BSI, bloodstream infection; CI, confidence interval; JBI, Joanna Briggs Institute; OR, odds ratio; PI, prediction interval; ROBINS-I, Risk Of Bias In Non-randomized Studies of Interventions; VV, veno-venous.

## Data Availability

The original contributions presented in this study are included in the article/Supplementary Material. Further inquiries can be directed to the corresponding author.

## Supplementary Materials

The following supporting information can be downloaded at: https://www.mdpi.com/article/10.3390/medsci14060653/s1, Table S1: PRISMA 2020 checklist; Table S2: Operational BSI case definition, source and tier for each report; Table S3: Database search strategies; Table S4: Full-text reports excluded, with reasons, at the eligibility and source-verification stages; Figure S1: Funnel plot and small-study tests for Outcome A; Figure S2: JBI prevalence checklist, item-level judgments for the 47 reports.

## Abbreviations

ARDS, acute respiratory distress syndrome; BSI, bloodstream infection; CAPA, COVID-19-associated pulmonary aspergillosis; CI, confidence interval; CRRT, continuous renal replacement therapy; ECMO, extracorporeal membrane oxygenation; ECPR, extracorporeal cardiopulmonary resuscitation; ELSO, Extracorporeal Life Support Organization; GLMM, generalized linear mixed model; GRADE, Grading of Recommendations Assessment, Development and Evaluation; HR, hazard ratio; ICU, intensive care unit; IQR, interquartile range; JBI, Joanna Briggs Institute; LCBI, laboratory-confirmed bloodstream infection; mNGS, metagenomic next-generation sequencing; NA, not applicable; NR, not reported; OR, odds ratio; PI, prediction interval; REML, restricted maximum likelihood; ROBINS-I, Risk Of Bias In Non-randomized Studies of Interventions; VA, veno-arterial; VAP, ventilator-associated pneumonia; VV, veno-venous.
