# Supplementary material for: Infectious Complications in Adults Receiving Extracorporeal Membrane Oxygenation: A Systematic Review and Meta-Analysis of Nosocomial and Bloodstream Infection, Prevalence, Risk Factors, and Clinical Outcomes
Source: Med Sci (Basel). 2026 Oct 9;14(6):653. doi: 10.3390/medsci14060653 (PMC13707242; doi:10.3390/medsci14060653)
Supplement: Supplementary file 1 [file medsci-14-00653-s001.zip › medsci-4586623-supplementary.pdf]

PRISMA

Table S1. PRISMA 2020 checklist. Section, table and figure numbers refer to the revised manuscript.

Section and Topic	Item #	Checklist item	Location where item is reported
**TITLE**			
Title	1	Identify the report as a systematic review.	Title
**ABSTRACT**			
Abstract	2	See the PRISMA 2020 for Abstracts checklist.	Abstract (structured per PRISMA 2020 for Abstracts: objectives, eligibility, information sources, risk-of-bias and certainty methods, synthesis methods, included reports, pooled results, limitations, interpretation; PROSPERO CRD420261442366)
**INTRODUCTION**			
Rationale	3	Describe the rationale for the review in the context of existing knowledge.	Section 1 (Introduction), paragraphs 1–5
Objectives	4	Provide an explicit statement of the objective(s) or question(s) the review addresses.	Section 1, final paragraph; Section 2.1 (population, exposure, comparator and outcomes)
**METHODS**			
Eligibility criteria	5	Specify the inclusion and exclusion criteria for the review and how studies were grouped for the syntheses.	Section 2.2 (inclusion and exclusion criteria, including the age and separability conditions applied during source verification); grouping for the syntheses in Sections 2.7 (Tier A–C case-definition classification) and 2.9 (eligibility rules for Outcomes A and B)
Information sources	6	Specify all databases, registers, websites, organisations, reference lists and other sources searched or consulted to identify studies. Specify the date when each source was last searched or consulted.	Section 2.3 and Supplementary Table S4: PubMed/MEDLINE, Embase, Scopus and Cochrane CENTRAL, searched from inception, with the date each database was last searched given in Table S4; Section 2.1, deviation (i) (Scopus added to the record.
Search strategy	7	Present the full search strategies for all databases, registers and websites, including any filters and limits used.	Supplementary Table S4: full search string for each database, exactly as run, with the number of records retrieved; Section 2.3. No search filters were used; publication-date and language limits were applied at screening.
Selection process	8	Specify the methods used to decide whether a study met the inclusion criteria of the review, including how many reviewers screened each record and each report retrieved, whether they worked independently, and if applicable, details of automation tools used in the process.	Section 2.4: two reviewers independently screened titles and abstracts, and a third arbitrated discordant judgments; full-text reports were assessed in two stages (eligibility; source verification), with all full-text exclusions re-examined during revision.
Data collection process	9	Specify the methods used to collect data from reports, including how many reviewers collected data from each report, whether they worked independently, any processes for obtaining or confirming data from study investigators, and if applicable, details of automation tools used in the process.	Section 2.6: data abstracted into a piloted 27-variable form; discrepancies resolved by consensus, with adjudication by a third reviewer. During revision, ECMO indication and antimicrobial prophylaxis were added and every value used in a table or pooled analysis was re-audited against the primary publication; corrections are recorded in the data-lock workbook
Data items	10a	List and define all outcomes for which data were sought. Specify whether all results that were compatible with each outcome domain in each study were sought (e.g. for all measures, time points, analyses), and if not, the methods used to decide which results to collect.	Sections 2.1 and 2.9: infection prevalence/incidence, pathogen distribution, risk factors, in-hospital and ICU mortality, ICU and hospital length of stay, ECMO duration, weaning/decannulation success. Mortality harmonized to the in-hospital end point (survival to discharge as its complement; mortality without a stated time frame accepted as reported); reports giving only ICU, on-ECMO, one-year or infected-only mortality excluded from that summary.

Section and Topic	Item #	Checklist item	Location where item is reported
			Outcome A exposure and end point recorded per study (Table 3).
	10b	List and define all other variables for which data were sought (e.g. participant and intervention characteristics, funding sources). Describe any assumptions made about any missing or unclear information.	Section 2.6 (27 variables: design, ECMO mode, demographics, case definition and window, microbiology, infection sites, ECMO duration, length of stay, mortality and end point, weaning, risk factors, predictors; ECMO indication and prophylaxis added at revision). Missing or unclear information: Section 2.9 and Table 1 notes (numeric/NR/NA notation; derived values marked †; episode-based numerators marked [e]).
Study risk of bias assessment	11	Specify the methods used to assess risk of bias in the included studies, including details of the tool(s) used, how many reviewers assessed each study and whether they worked independently, and if applicable, details of automation tools used in the process.	Section 2.8: ROBINS-I at the level of the result for all 37 comparative results; JBI prevalence checklist for all 47 reports, with operational rules for items 3, 6 and 7; ratings by one reviewer, verified by a second, disagreements resolved by discussion.
Effect measures	12	Specify for each outcome the effect measure(s) (e.g. risk ratio, mean difference) used in the synthesis or presentation of results.	Section 2.9: Outcome A, crude odds ratio (infected vs uninfected) from reconstructed 2 × 2 tables; Outcome B, patient-level proportion (pooled on the logit scale); descriptive outcomes as median, IQR and range of study-level values.
Synthesis methods	13a	Describe the processes used to decide which studies were eligible for each synthesis (e.g. tabulating the study intervention characteristics and comparing against the planned groups for each synthesis (item #5)).	Sections 2.7 and 2.9: Outcome A, every study with a reconstructable 2 × 2 mortality table (24 of 47); Outcome B, Tier A definition plus patient-level numerator plus matching cohort denominator (7 of 47); seven reports narrative only. Role of each report shown in Table 1 (Pooled role column).
	13b	Describe any methods required to prepare the data for presentation or synthesis, such as handling of missing summary statistics, or data conversions.	Sections 2.5 and 2.9: patient- and run-level denominators kept separate; episode-based numerators excluded from patient-level prevalence; hours converted to days; mortality harmonized; 2 × 2 tables reconstructed from reported percentages or strata where necessary (Table 3 footnotes); derived values and NR/NA notation defined in Table 1 notes.
	13c	Describe any methods used to tabulate or visually display results of individual studies and syntheses.	Section 2.9; Table 1 (study characteristics), Table 2 (BSI by tier), Tables 3 and 4 (study-level data for Outcomes A and B), Figures 2 and 3 (forest plots with weights and prediction intervals), Figure 4 (ROBINS-I), Supplementary Figures S1 (funnel plot) and S2 (JBI).
	13d	Describe any methods used to synthesize results and provide a rationale for the choice(s). If meta-analysis was performed, describe the model(s), method(s) to identify the presence and extent of statistical heterogeneity, and software package(s) used.	Sections 2.9 and 2.10: Outcome A, random-effects model with REML τ^2 and Hartung–Knapp CIs, 95% prediction interval; Outcome B, logistic-normal GLMM (maximum likelihood) with profile-likelihood CI check; rationale stated; heterogeneity by Cochran's Q, I^2, τ^2 (τ for Outcome B) and prediction intervals; registered DerSimonian–Laird, Mantel–Haenszel and Freeman–Tukey models reported for comparison; R 4.3.3, metafor 4.4-0, lme4 1.1-35.1.
	13e	Describe any methods used to explore possible causes of heterogeneity among study results (e.g. subgroup analysis, meta-regression).	Section 2.9: exploratory subgroup analyses by exposure construct, ECMO indication and COVID-19 cohort status (Outcome A); COVID-19 subgroup analysis and GLMM meta-regression (Outcome B); added post hoc (Section 2.1, deviation vi).
	13f	Describe any sensitivity analyses conducted to assess robustness of the synthesized results.	Section 2.9: Outcome A, BSI-only exposure, in-hospital end point, patient-level only, age-compliant cohorts, exclusion of the fungal exposure, the selected-sample outlier, the influential study and

PRISMA

Section and Topic	Item #	Checklist item	Location where item is reported
			reconstructed tables, addition of two studies, leave-one-out and influence diagnostics; Outcome B, exclusion or substitution of Pieri 2012 and addition of Sun 2010.
Reporting bias assessment	14	Describe any methods used to assess risk of bias due to missing results in a synthesis (arising from reporting biases).	Section 2.9: Outcome A, Egger's regression, Begg's rank correlation, Peters' test, contour-enhanced funnel plot and trim-and-fill (L0 and R0); Outcome B, not assessed (k = 7; Section 2.1, deviation vii).
Certainty assessment	15	Describe any methods used to assess certainty (or confidence) in the body of evidence for an outcome.	Section 2.8: GRADE; ROBINS-I-assessed evidence and event rates started at high certainty (deviation ix); each limitation counted in one domain only.
**RESULTS**			
Study selection	16a	Describe the results of the search and selection process, from the number of records identified in the search to the number of studies included in the review, ideally using a flow diagram.	Sections 2.4 and 3.1; Figure 1: 6,896 records, 367 duplicates, 6,529 screened, 61 full texts assessed, 9 excluded at eligibility and 5 at source verification, 47 reports (46 cohorts) included.
	16b	Cite studies that might appear to meet the inclusion criteria, but which were excluded, and explain why they were excluded.	Supplementary Table S3: 14 excluded full-text reports, with stage and reason; Section 2.4.
Study characteristics	17	Cite each included study and present its characteristics.	Section 3.1; Table 1 (47 reports with characteristics and detail rows); Supplementary Table S2 (case definition and tier for each report).
Risk of bias in studies	18	Present assessments of risk of bias for each included study.	Section 3.11; Figure 4 (ROBINS-I, domain and overall judgments for 37 comparative results and two additional adjusted results); Supplementary Figure S2 (JBI item-level judgments for 47 reports).
Results of individual studies	19	For all outcomes, present, for each study: (a) summary statistics for each group (where appropriate) and (b) an effect estimate and its precision (e.g. confidence/credible interval), ideally using structured tables or plots.	Table 3 (Outcome A: deaths/n by group, crude OR with 95% CI for 24 studies); Table 4 (Outcome B: BSI/patients with exact 95% CI for 7 studies); Figures 2 and 3; Table 1 (study-level BSI proportion and mortality).
Results of syntheses	20a	For each synthesis, briefly summarise the characteristics and risk of bias among contributing studies.	Sections 3.9–3.11; Tables 3 and 4 (exposure, end point, mode and COVID-19 status of contributing studies); Figure 4 and Supplementary Figure S2.
	20b	Present results of all statistical syntheses conducted. If meta-analysis was done, present for each the summary estimate and its precision (e.g. confidence/credible interval) and measures of statistical heterogeneity. If comparing groups, describe the direction of the effect.	Sections 3.9 and 3.10; Figures 2 and 3; Tables 3 and 4. Outcome A: OR 1.65 (95% CI 1.33–2.04), I^2 34%, τ^2 0.059, PI 0.95–2.85 (24 studies). Outcome B: 25.8% (95% CI 17.3–36.5%), τ (logit) 0.63, I^2 90.3%, PI 5.7–66.5% (7 studies). Descriptive summaries in Sections 3.2–3.8 and Table 2.
	20c	Present results of all investigations of possible causes of heterogeneity among study results.	Section 3.9 (subgroups by exposure construct, indication and COVID-19 status; influence diagnostics) and Section 3.10 (COVID-19 subgroup and meta-regression).
	20d	Present results of all sensitivity analyses conducted to assess the robustness of the synthesized results.	Sections 3.9 and 3.10 (all sensitivity analyses, with registered-model estimates); Table 5 footnotes.
Reporting biases	21	Present assessments of risk of bias due to missing results (arising from reporting biases) for each synthesis assessed.	Section 3.9; Supplementary Figure S1. Outcome A: Egger p = 0.002 (REML-based variant p = 0.004; p = 0.062 without Nesseler 2024); Begg p = 0.29; Peters p = 0.21; trim-and-fill OR 1.38 (1.08–1.76; L0, nine studies imputed) and 1.46 (1.13–1.87; R0). Outcome

Section and Topic	Item #	Checklist item	Location where item is reported
			B: not tested (k = 7).
Certainty of evidence	22	Present assessments of certainty (or confidence) in the body of evidence for each outcome assessed.	Section 3.12; Table 5 (GRADE evidence profile with absolute effects): very low certainty for both outcomes.
**DISCUSSION**			
Discussion	23a	Provide a general interpretation of the results in the context of other evidence.	Section 4 (Discussion)
	23b	Discuss any limitations of the evidence included in the review.	Section 4, limitations paragraph (observational evidence at serious risk of bias, definitional heterogeneity, case mix, units of analysis, missing long-term outcomes)
	23c	Discuss any limitations of the review processes used.	Section 4, limitations paragraph (no prospective registration, post hoc methodological choices, reports reinstated on re-audit, English-language restriction)
	23d	Discuss implications of the results for practice, policy, and future research.	Section 4 (stewardship, reporting standards and trial designs) and Section 5 (Conclusions)
**OTHER INFORMATION**			
Registration and protocol	24a	Provide registration information for the review, including register name and registration number, or state that the review was not registered.	Abstract; Section 2.1; reference 17: PROSPERO CRD420261442366, registered 6 July 2026 (not prospectively registered).
	24b	Indicate where the review protocol can be accessed, or state that a protocol was not prepared.	Section 2.1: registration record and amended versions at https://www.crd.york.ac.uk/PROSPERO/view/CRD420261442366; the full protocol was uploaded to PROSPERO at registration.
	24c	Describe and explain any amendments to information provided at registration or in the protocol.	Section 2.1, deviations (i)–(x), each with rationale and cross-reference to the affected section; recorded in PROSPERO versions 2.0 and 3.0 (1 October 2026); registered-method estimates reported alongside (Sections 3.9 and 3.10).
Support	25	Describe sources of financial or non-financial support for the review, and the role of the funders or sponsors in the review.	No fund was received
Competing interests	26	Declare any competing interests of review authors.	Conflicts of Interest statement: Non
Availability of data, code and other materials	27	Report which of the following are publicly available and where they can be found: template data collection forms; data extracted from included studies; data used for all analyses; analytic code; any other materials used in the review.	Data Availability Statement and Section 2.6: extracted data are presented in Tables 1–4 and Supplementary Tables S2–S3; the data-lock workbook is available from the corresponding author on request.

From: Page MJ, McKenzie JE, Bossuyt PM, Boutron I, Hoffmann TC, Mulrow CD, et al. The PRISMA 2020 statement: an updated guideline for reporting systematic reviews. BMJ 2021;372:n71. doi: 10.1136/bmj.n71. This work is licensed under CC BY 4.0. To view a copy of this license, visit https://creativecommons.org/licenses/by/4.0/

Supplementary Table S2. Operational bloodstream infection case definition, source and tier for each of the 47 included reports

Report	Definition cited by the authors	Operational criterion applied	Ascertainment window	Unit of analysis	Commensal rule	Tier	Basis for tier and flags	Outcome B
**Andersen 2023 [20]**	Institutional / culture-based	Bacteremia = growth of bacteria in blood culture + fever and/or raised CRP and/or raised PCT. No CoNS contaminant adjudication (CoNS 11/50 = 22% of the cohort). Not CDC/NHSN.	During ECMO	Patients	No	B	Culture-based; no two-culture rule for common commensals	No (not Tier A)
**Andreev 2023 [21]**	Institutional / culture-based	Blood cultures drawn only when bacteremia/fungemia clinically suspected. No formal case definition; no contaminant adjudication (CoNS counted).	During ECMO	Patients	No	B	Culture-based; no two-culture rule for common commensals	No (not Tier A)
**Aubron 2013 [9]**	CDC/NHSN	CDC/NHSN modified; >48h after ECMO initiation and ≤72h post-decannulation	During ECMO	Procedures	Yes	A	LCBI logic applied. Analyzed by procedure	No (procedure/run unit)
**Boscolo 2024 [22]**	MDR GN surveillance-based; BSI definitions in supplement	Primary exposure is MDR Gram-negative bacteria detected by routine surveillance, which includes colonization without clinical signs of infection. BSI/CR-BSI definitions are relegated to the supplement and not quantified in the main text.	Pre- and during ECMO	Patients	NA	NA	Not assigned: Exposure is MDR Gram-negative carriage detected by surveillance, including colonization; BSI not quantified in the main report	No (no tier)
**Carelli 2023 [23]**	LCBI-consistent (CDC/NHSN 2022 cited)	De novo laboratory-confirmed BSI; ≥1 peripheral blood-culture set for a recognized pathogen; ≥2 peripheral sets for common skin commensals (CoNS, Corynebacterium, Bacillus); cultures drawn on clinical or biological signs of sepsis; primary vs secondary BSI (same pathogen at another sterile site within 24 h)	48 h after ECMO start to 48 h after decannulation	Patients	Yes	A	LCBI logic applied	Primary
**Cavayas 2018 [24]**	Other / unclassified	ICD-9 coded fungal infection or any positive fungal culture	During ECMO	Patients	NA	NA	Not assigned: Registry-coded fungal infection at any site; sample selected on infection status	No (no tier)
**Gao 2023 [25]**	MDR-focused (Magiorakos); not a BSI case definition	Cohort split into MDR-infection group (R) vs non-MDR group (N). MDR identification per 'proposed amendments regarding the definitions of MDR and XDR pathogens'. No BSI-specific case definition.	During and post-ECMO	Patients	NA	NA	Not assigned: MDR organism isolation (Magiorakos criteria); not a BSI case definition	No (no tier)
**Garcia 2013 [26]**	Other / unclassified	Aspergillus isolation; invasive in 1 case (autopsy)	During ECMO	Patients	NA	NA	Not assigned: Registry-coded Aspergillus isolation, which may include colonization; sample selected on infection status	No (no tier)
**Grasselli 2017 [8]**	Guideline-based adjudication (ATS/IDSA)	All positive cultures adjudicated by two intensivists and two ID specialists against ATS/IDSA 2005 (VAP), IDSA 2010 (CAUTI) and IDSA 2009 (catheter infection) guidelines; detailed criteria in the source report’s online supplement (not available); only the first NI episode analyzed	ECMO start to 48 h after decannulation	Patients	No	B	Culture-based; no two-culture rule for common commensals. Provisional: detailed criteria are in an online supplement of the source that was not available	No (not Tier A)

Report	Definition cited by the authors	Operational criterion applied	Ascertainment window	Unit of analysis	Commensal rule	Tier	Basis for tier and flags	Outcome B
**Haneke 2016 [19]**	CDC/NHSN	CDC 1988 criteria; infection >48h after ECMO initiation	During ECMO	Patients	No	B	Culture-based; no two-culture rule for common commensals. CDC/NHSN cited; commensal rule not applied; cohort includes ECLS and pECLA	No (not Tier A)
**Hsu 2009 [27]**	CDC/NHSN	CDC/NHSN definitions (24h post-ECMO to 48h post-decannulation)	During ECMO	Patients	No	B	Culture-based; no two-culture rule for common commensals	No (not Tier A)
**Joshi 2024 [28]**	CDC/NHSN	Surviving Sepsis Guidelines + NHSN + microbiological criteria	During ECMO	Patients; BSI numerator episode-based	Yes (by adoption, not restated)	A	LCBI logic applied. Tier A by explicit adoption of NHSN LCBI criteria (commensal rule not restated); episode-based numerator	No (episode-based numerator)
**Juthani 2018 [29]**	CDC/NHSN	CDC definition, >48h after ECMO or ≤3 days post-decannulation	During ECMO	Runs	No	B	Culture-based; no two-culture rule for common commensals	No (not Tier A)
**Kang 2025 [30]**	mNGS-based composite (not CDC/NHSN)	No blood-culture-based CDC/NHSN criterion. Ascertainment conditional on temperature >38.5C.	During ECMO	Patients	NA	C	Non-culture ascertainment. Metagenomic next-generation sequencing composite without a culture criterion	No (not Tier A)
**Kim 2016 [31]**	Institutional / culture-based	Positive blood culture >48h after ECMO initiation to 24h post-decannulation	During ECMO	Patients	No	B	Culture-based; no two-culture rule for common commensals. Catheter-tip culture study; sample restricted to patients with tip culture	No (not Tier A)
**Kim 2017 [32]**	CDC/NHSN	CDC/NHSN criteria; ≥48h after ECMO until 24h post-weaning	During ECMO	Patients	No	B	Culture-based; no two-culture rule for common commensals	No (not Tier A)
**Kızmaz 2023 [33]**	CDC 1988 (Garner/Hughes) + clinical status	NI 24 h after initiation to 48 h after discontinuation; diagnosis per CDC 1988 definitions (ref 13) OR patient's clinical status. not modern CDC/NHSN LCBI.	During ECMO	Patients	No	B	Culture-based; no two-culture rule for common commensals. CDC 1988 criteria predate the NHSN LCBI framework	No (not Tier A)
**Ko 2020 [34]**	CDC	NIs defined per CDC definitions (ref 18); only each patient's FIRST episode of NI included.	During ECMO	Patients	No	B	Culture-based; no two-culture rule for common commensals	No (not Tier A)
**Lee 2022 [35]**	Institutional / culture-based	No CDC/NHSN citation.	During and post-ECMO	Patients	No	B	Culture-based; single commensal culture counted as BSI when clinical signs are present.	No (not Tier A)
**Li 2023 [36]**	China NHC 2001 (derived from CDC 1988) — not CDC/NHSN LCBI	BSI = clinical manifestation (fever >38C, chills or hypotension) + positive blood culture from one or more blood samples. No skin-commensal exclusion rule → more permissive than LCBI; inflates BSI.	During and post-ECMO	Patients; BSI numerator episode-based	No	B	Culture-based; no two-culture rule for common commensals. China NHC 2001 criteria, derived from CDC 1988; exposure includes infections after decannulation	No (not Tier A)
**Menaker 2019 [37]**	CDC/NHSN	CDC/NHSN criteria: 2+ positive cultures + symptoms	During ECMO	Patients	No	B	Culture-based; no two-culture rule for common commensals	No (not Tier A)

Report	Definition cited by the authors	Operational criterion applied	Ascertainment window	Unit of analysis	Commensal rule	Tier	Basis for tier and flags	Outcome B
**Na 2018 [38]**	CDC/NHSN	LCBI criteria: recognized pathogen from blood culture, OR clinical manifestation + skin commensal from ≥2 cultures. Window >48 h after initiation to decannulation.	During ECMO	Patients	Yes	A	LCBI logic applied	Primary
**Nesseler 2024 [39]**	EUROBACT / LCBI-equivalent (not a CDC/NHSN citation)	BSI = positive blood culture ≥48 h after admission; skin commensals require 2 cultures from separate puncture sites. Cites ref 14 = Tabah (EUROBACT), not CDC/NHSN.	During ECMO	Patients	Yes	A	LCBI logic applied. EUROBACT criteria (LCBI-equivalent); BSI window anchored to ICU admission rather than cannulation	Primary
**Ortuño 2025 [40]**	ECMO cannula-related infection (ECMO-CRI)	ECMO-CRI = clinical suspicion PLUS a positive bacterial sample from the ECMO cannulation site.	During ECMO	Patients	NA	NA	Not assigned: Cannula-related infection case series; no at-risk denominator	No (no tier)
**Pieri 2012 [41]**	CDC/NHSN	CDC criteria + microbiology	Before and during ECMO	Patients	Yes (by adoption, not restated)	A	LCBI logic applied. Tier A by explicit adoption of NHSN LCBI criteria (commensal rule not restated); case–control sampling	Primary
**Pieri 2013 [42]**	Institutional / culture-based	Positive culture from any site (blood, BAL, urine). No BSI-specific criteria.	During ECMO	Patients	No	B	Culture-based; no two-culture rule for common commensals. Later report of the Pieri 2012 cohort; institutional culture-based definition	Sensitivity
**Pluim 2012 [43]**	ELSO registry-coded	Registry infection coding, all ages; fungal-timing strata.	Before and during ECMO	Patients	NA	NA	Not assigned: Registry-coded fungal infection (ELSO); BSI not quantified separately	No (no tier)
**Poth 2022 [44]**	Consensus criteria (fungal)	Candida from sterile sites = proven IFD (EORTC/MSG 2020); Candida in tracheal aspirate, BAL or urine = colonization; Aspergillus = any culture from respiratory fluids with clinical features (does not meet consensus criteria)	While on ECMO support	Patients	NA	NA	Not assigned: Invasive fungal disease by consensus criteria; only candidemia quantified as BSI	No (no tier)
**Poth 2024 [45]**	Consensus criteria (fungal)	CDC/EORTC/ESICM consensus, IC & pIPA criteria applied	During ECMO	Patients	NA	NA	Not assigned: Invasive fungal disease by consensus criteria; only candidemia quantified as BSI	No (no tier)
**Quintana 2021 [46]**	Institutional / culture-based (not CDC/NHSN)	'Bloodstream infection was defined by positive blood cultures with a pathogenic organism.' No commensal 2-culture rule. CLABSI is defined separately and idiosyncratically ('within 48 h of central line placement').	During and post-ECMO	Patients	No	B	Culture-based; no two-culture rule for common commensals	No (not Tier A)
**Schmidt 2012 [10]**	CDC/NHSN (NNIS 1988)	CDC NNIS definitions (Garner 1988); ECMO-associated = onset >24 h after ECMO initiation to 48 h after discontinuation; BSI catheter-related when the same organism is recovered from blood and catheter tip; >2 positive blood cultures required for coagulase-negative staphylococci; blood cultures drawn for temperature >38 °C or <36 °C or signs of sepsis	>24 h after ECMO initiation to 48 h after discontinuation	Patients	Yes	A	LCBI logic applied	Primary
**Schweickhardt 2023 [47]**	Institutional / culture-based	Positive culture + treated with antibiotics	Post-ECMO	Patients	NA	NA	Not assigned: Denominator restricted to infected patients	No (no tier)

Report	Definition cited by the authors	Operational criterion applied	Ascertainment window	Unit of analysis	Commensal rule	Tier	Basis for tier and flags	Outcome B
**Selçuk 2021 [48]**	CDC 1988 (Garner)	CDC 1988 (Garner) nosocomial infection criteria; ECMO-related infection = onset from 24 h after ECMO initiation to 48 h after ECMO end; no commensal rule stated	24 h after initiation to 48 h after end of ECMO	Patients	No	B	Culture-based; no two-culture rule for common commensals. CDC 1988 criteria predate the NHSN LCBI framework; adult subgroup only	No (not Tier A)
**Silvetti 2019 [49]**	Institutional / culture-based	New positive blood culture 48 h after ECMO initiation to 48 h after discontinuation. Not CDC/NHSN.	During ECMO	Patients	No	B	Culture-based; no two-culture rule for common commensals	No (not Tier A)
**Sun 2010 [11]**	CDC/NHSN	CDC criteria; nosocomial during ECMO only	During ECMO	Patients	No	B	Culture-based; no two-culture rule for common commensals. Reclassified from Tier A at re-audit: a single commensal culture with clinical signs was counted as BSI	Sensitivity
**Suzuki 2023 [50]**	Institutional / culture-based	Culture-proven infection during ECMO	During ECMO	Runs	NA	NA	Not assigned: Registry-coded culture-positive infection at any site	No (no tier)
**Sysiak-Sławecka 2023 [51]**	Institutional / culture-based	New infection (bacteremia, VAP, UTI) during ICU stay, clinical and culture-based	During ECMO	Patients	NA	NA	Not assigned: BSI window is the ICU stay, not ECMO support	No (no tier)
**Thomas 2017 [52]**	CDC/NHSN	CDC and French catheter infection guidelines adapted for ECMO	At ECMO removal (device cultures)	Runs	Yes	A	LCBI logic applied. Device-related BSI with paired cannula and blood cultures; analyzed by run	No (procedure/run unit)
**Uçar 2024 [53]**	CDC/NHSN	CDC/NHSN (Horan 2008); ECMO-related = diagnosed after the first 48 h of cannulation up to 48 h after decannulation; blood cultures from two different sites on fever ≥37.5 °C, leukocytosis or raised CRP/PCT	48 h after cannulation to 48 h after decannulation	Patients	Yes	A	LCBI logic applied	Primary
**Wang 2020 [54]**	CDC 1988 (Garner) implicit — no explicit BSI criteria	Ref 7 = Garner 1988 CDC definitions, cited ONLY for the nosocomial pneumonia definition. No BSI/LCBI criteria stated anywhere.	During ECMO	Patients	No	B	Culture-based; no two-culture rule for common commensals. CDC 1988 criteria implied; no explicit BSI criteria	No (not Tier A)
**Wang 2021 [55]**	CDC/NHSN	CDC definitions; occurring ≥24h after ECMO initiation and ≤48h post-decannulation	During ECMO	Patients; BSI numerator episode-based	No	B	Culture-based; no two-culture rule for common commensals	No (not Tier A)
**Wang 2023 [18]**	Institutional / culture-based	≥24h post-ECMO initiation to ≤48–72h post-weaning (RT-NISS + clinician confirmation)	During ECMO	Patients	NA	NA	Not assigned: Nosocomial infection by institutional criteria; BSI counted as episodes only	No (no tier)
**Wells 2025 [56]**	CoNS-only ascertainment; study proposes a definition	The paper's purpose is definitional: it proposes that CoNS bacteremia require isolation from 2 subsequent blood cultures. Only 5/13 (38%) met that bar.	During ECMO	Patients	NA	NA	Not assigned: Ascertains coagulase-negative staphylococci only; the study proposes a definition	No (no tier)

Report	Definition cited by the authors	Operational criterion applied	Ascertainment window	Unit of analysis	Commensal rule	Tier	Basis for tier and flags	Outcome B
**Xu 2022 [57]**	CDC/NHSN	BSI = ≥1 positive blood culture for an identified pathogen, OR 2 positive cultures within 48 h for a common skin contaminant plus symptoms. LCBI-consistent.	During ECMO	Patients; BSI numerator episode-based	Yes	A	LCBI logic applied. Episode-based numerator	No (episode-based numerator)
**Yang 2022 [58]**	CDC/NHSN	Explicitly per CDC/NHSN definitions: two separate blood cultures positive for the same organism + signs of infection; named skin commensals require 2 cultures within 48 h. Window 24 h after ECMO to 48 h after withdrawal.	During ECMO	Patients	Yes	A	LCBI logic applied	Primary
**Yun 2021 [59]**	Institutional / culture-based	BSI 2 d after ECMO initiation to 2 d after removal	During ECMO	Patients	No	B	Culture-based; no two-culture rule for common commensals	No (not Tier A)
**Zhao 2025 [60]**	CDC/NHSN-referenced	Definitions referenced to Spalding/Zingg conventions; BSI not separately quantified at patient level.	During ECMO	Patients	No	B	Culture-based; no two-culture rule for common commensals	No (not Tier A)

Abbreviations. ATS/IDSA, American Thoracic Society/Infectious Diseases Society of America; BAL, bronchoalveolar lavage; BSI, bloodstream infection; CAUTI, catheter-associated urinary tract infection; CDC, Centers for Disease Control and Prevention; CoNS, coagulase-negative staphylococci; CR-BSI, catheter-related BSI; CRP, C-reactive protein; ECD, extracorporeal device; ECLS, extracorporeal life support; ECMO, extracorporeal membrane oxygenation; ECMO-AI, ECMO-associated infection; ELSO, Extracorporeal Life Support Organization; EORTC/MSG, European Organisation for Research and Treatment of Cancer/Mycoses Study Group; EUROBACT, European bacteremia study criteria; ICU, intensive care unit; IDSA, Infectious Diseases Society of America; LCBI, laboratory-confirmed bloodstream infection; MDR, multidrug-resistant; mNGS, metagenomic next-generation sequencing; NHC, National Health Commission (China); NHSN, National Healthcare Safety Network; NI, nosocomial infection; NNIS, National Nosocomial Infections Surveillance; NR, not reported; PCT, procalcitonin; pECLA, pumpless extracorporeal lung assist; VA, venoarterial; VAP, ventilator-associated pneumonia; VV, venovenous. Reference numbers correspond to the main-text reference list.

Table S3. Database search strategies

**Database**	**Search string**
PubMed/MEDLINE	("Extracorporeal Membrane Oxygenation"[Mesh] OR ECMO[tiab] OR "extracorporeal life support"[tiab]) AND ("Cross Infection"[Mesh] OR "nosocomial infection*"[tiab] OR "healthcare-associated infection*"[tiab] OR "bloodstream infection*"[tiab] OR bacteremia[tiab] OR bacteraemia[tiab] OR pneumonia[tiab] OR fungemia[tiab] OR fungaemia[tiab] OR candidemia[tiab] OR candidaemia[tiab])
Scopus	("extracorporeal membrane oxygenation" OR ECMO OR "extracorporeal life support") AND ("nosocomial infection*" OR "healthcare-associated infection*" OR "hospital-acquired infection*" OR "bloodstream infection*" OR bacter?emia OR pneumonia OR fung?emia OR candid?emia)
Embase	Extracorporeal membrane oxygenation AND Infection
Cochrane CENTRAL	("extracorporeal membrane oxygenation" OR ECMO OR "extracorporeal life support") AND ((nosocomial NEXT infection*) OR (healthcare NEXT associated NEXT infection*) OR (hospital NEXT acquired NEXT infection*) OR (bloodstream NEXT infection*) OR bacteremia OR bacteraemia OR pneumonia OR fungemia OR fungaemia OR candidemia OR candidaemia)

Supplementary Table S4. Full-text reports excluded, with reasons, at the eligibility and source-verification stages (n = 14)

No.	Report	Full citation	DOI	Reason for exclusion
**A. Full-text eligibility stage (n = 9)**				
1	**Ait Hssain 2024**	Ait Hssain A, Vahedian-Azimi A, Ibrahim AS, Hassan IF, Azoulay E, Darmon M. Incidence, risk factors and outcomes of nosocomial infection in adult patients supported by extracorporeal membrane oxygenation: a systematic review and meta-analysis. Crit Care. 2024;28(1):158.	10.1186/s13054-024-04946-8	Systematic review and meta-analysis (not primary data) [a]
2	**Lv 2024**	Lv X, Han Y, Liu D, Chen X, Chen L, Huang H, Huang C. Risk factors for nosocomial infection in patients undergoing extracorporeal membrane oxygenation support treatment: a systematic review and meta-analysis. PLoS ONE. 2024;19(11):e0308078.	10.1371/journal.pone.0308078	Systematic review and meta-analysis (not primary data) [a]
3	**Kim 2021**	Kim JH, Pieri M, Landoni G, Scandroglio AM, Calabrò MG, Fominskiy E, Lembo R, Heo MH, Zangrillo A. Venovenous ECMO treatment, outcomes, and complications in adults according to large case series: a systematic review. Int J Artif Organs. 2021;44(7):481–488.	10.1177/0391398820975408	Systematic review of VV-ECMO case series (not primary data)
4	**Marcus 2024**	Marcus JE, Shah A, Peek GJ, MacLaren G. Nosocomial infections in adults receiving extracorporeal membrane oxygenation: a review for infectious diseases clinicians. Clin Infect Dis. 2024;79(2):412–419.	10.1093/cid/ciae120	Narrative review (not primary data) [a]
5	**Jain 2022**	Jain A, Mehta Y. Sepsis associated with extracorporeal membrane oxygenation. J Card Crit Care TSS. 2022;6(2):146–150.	10.1055/s-0042-1757392	Narrative review (not primary data)
6	**Corley 2019**	Corley A, Lye I, Lavana JD, Ahuja A, Anstey CM, Jarrett P, Haisz E, Parke R, Pellegrino V, Buscher H, et al. Nosocomial infection prevalence in patients undergoing extracorporeal membrane oxygenation (ECMO): protocol for a point prevalence study across Australia and New Zealand. BMJ Open. 2019;9(7):e029293.	10.1136/bmjopen-2019-029293	Study protocol; no results reported
7	**Schilcher 2019**	Schilcher G, Eisner F, Hackl G, Eller P, Valentin T, Zollner-Schwetz I, Krause R, Brcic L. Candida infection of membrane oxygenator during ECMO therapy. J Infect. 2019;78(1):75–86.	10.1016/j.jinf.2018.08.011	Case report (letter); fewer than 10 patients
8	**O'Neill 2001**	O'Neill JM, Schutze GE, Heulitt MJ, Simpson PM, Taylor BJ. Nosocomial infections during extracorporeal membrane oxygenation. Intensive Care Med. 2001;27(8):1247–1253.	10.1007/s001340101029	Pediatric-predominant cohort (4 of 141 patients older than 18 years); adult data not separable
9	**Esposito 2021**	Esposito EC, Jones KM, Galvagno SM, Kaczorowski DJ, Mazzeffi MA, DiChiacchio L, Deatrick KB, Madathil RJ, Herrold JA, Rabinowitz RP, et al. Incidence of healthcare-associated infections in patients with fever during the first 48 hours after decannulation from veno-venous extracorporeal membrane oxygenation. Perfusion. 2021;36(4):421–428.	10.1177/0267659120948427	Infection not acquired during ECMO support: infections ascertained only in the 48 hours after decannulation, in survivors
**B. Source-verification stage, after data extraction (n = 5)**				
10	**Menekşe 2022**	Menekşe Ş, Deniz S. Secondary infections in COVID-19 patients: a two-centre retrospective observational study. J Infect Dev Ctries. 2022;16(8):1294–1301.	10.3855/jidc.15637	ECMO a covariate within a general COVID-19 ICU cohort, not the entry criterion

No.	Report	Full citation	DOI	Reason for exclusion
11	**Zhang 2021**	Zhang J, Lan P, Yi J, Yang C, Gong X, Ge H, Xu X, Liu L, Zhou J, Lv F. Secondary bloodstream infection in critically ill patients with COVID-19. J Int Med Res. 2021;49(12):3000605211062783.	10.1177/03000605211062783	ECMO a covariate within a general COVID-19 ICU cohort, not the entry criterion
12	**Huang 2019**	Huang L, Zhang N, Huang X, Xiong S, Feng Y, Zhang Y, Li M, Zhan Q. Invasive pulmonary aspergillosis in patients with influenza infection: a retrospective study and review of the literature. Clin Respir J. 2019;13(4):202–211.	10.1111/crj.12995	ECMO not the entry criterion; aspergillosis an influenza co-infection, not acquired during ECMO
13	**Hahne 2015**	Hahne K, Horstmann C, Fischer D, Köck R, Peters G, Lebiedz P. Cannula-related infection in adult medical intensive care unit patients undergoing extracorporeal life support and extracorporeal membrane oxygenation. J Hosp Infect. 2015;91(4):372–374.	10.1016/j.jhin.2015.08.022	ECMO population not separable from patients on other extracorporeal life support, including pumpless extracorporeal lung assist; cannula-related rather than bloodstream infection reported
14	**Noah 2010**	Noah MA, Dawrant M, Faulkner GM, Hill AM, Harvey C, Hussain A, Jenkins DR, Nichani S, Peek GJ, Sosnowski AW, et al. Panton-Valentine leukocidin expressing Staphylococcus aureus pneumonia managed with extracorporeal membrane oxygenation: experience and outcome. Crit Care Med. 2010;38(11):2250–2253.	10.1097/CCM.0b013e3181f26490	Case series of four patients; infection was the indication for ECMO, not a complication of it

Reconciliation with Figure 1. Database searches identified 6,896 records; 367 duplicates were removed and 6,529 records were screened on title and abstract, of which 6,468 were excluded. Sixty-one full-text reports were assessed. Nine were excluded at the full-text eligibility stage (band A), leaving 52 reports for data extraction and source verification; five of these were excluded at the source-verification stage (band B), leaving 47 included reports (61 − 9 − 5 = 47). The 47 reports describe 46 unique cohorts because Pieri 2012 and Pieri 2013 describe the same cohort. The 52 extracted reports include six that had been excluded at the full-text stage of the original screen and were reinstated when every exclusion was re-examined during revision (Carelli 2023, Grasselli 2017, Poth 2022, Schmidt 2012, Selçuk 2021 and Uçar 2024); they are included reports and therefore do not appear in this table.

Grounds for exclusion. Band A: not primary observational data (five reviews and one protocol), fewer than 10 patients (one case report), population not separable as adult (one) and exposure outside the ECMO window (one). Band B: ECMO not the entry criterion (three), ECMO population not separable from other extracorporeal support (one) and infection as the indication for, rather than a complication of, ECMO (one). Source-verification exclusions were applied after full extraction, when the full text showed that an eligibility condition was not met; the two conditions concerned (ECMO as the entry criterion and separability of the ECMO population) were added to the eligibility criteria at that stage and are declared as deviations from the registered protocol.

[a] Cited as background in the manuscript (Marcus 2024 [1], Ait Hssain 2024 [16], Lv 2024 [68]); excluded only as a source of primary data. Bibliographic details were taken from PubMed records, except Jain 2022 (not indexed in PubMed; publisher record). DOI, digital object identifier; ECMO, extracorporeal membrane oxygenation; ICU, intensive care unit; VV, venovenous.

Supplementary Figure S1. Funnel plot for Outcome A (mortality, infected vs non-infected; k = 24)

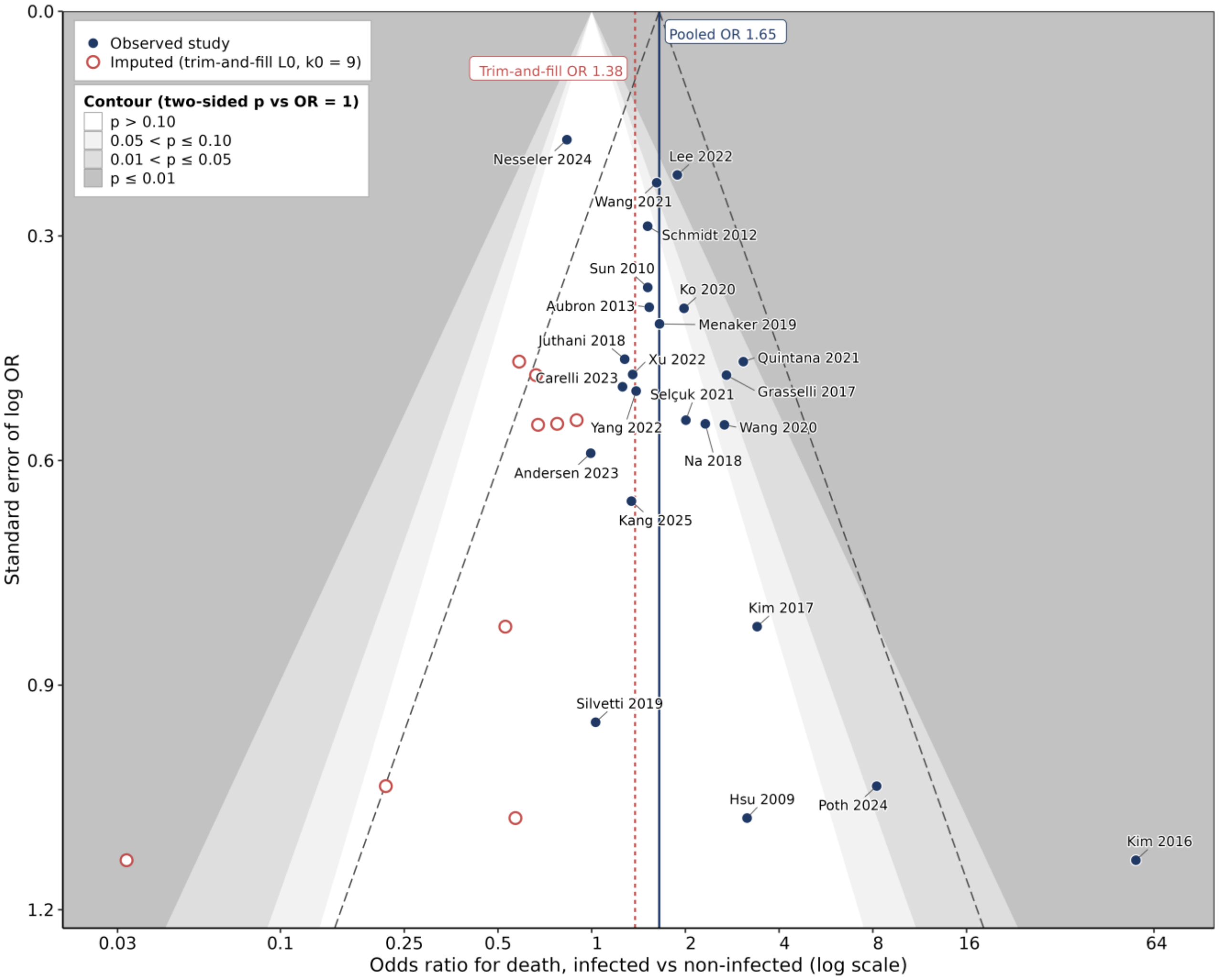

Shaded contours: statistical significance of individual studies against OR = 1. Dashed grey lines: pseudo-95% limits around the pooled estimate.
Pooled OR 1.65 (1.33-2.04), REML + Hartung-Knapp, k = 24. Egger test p = 0.002; Begg test p = 0.290. Trim-and-fill (L0, 9 imputed) OR 1.38 (1.08-1.76).

Supplementary Figure S2. JBI critical appraisal for prevalence studies across the 47 included reports

	Q1	Q2	Q3	Q4	Q5	Q6	Q7	Q8	Q9	Concern	Outcome B
Andersen 2023	+	?	×	+	+	?	?	+	+	Moderate	
Andreev 2023	+	?	×	+	+	×	×	×	+	High	
Aubron 2013	+	+	×	+	+	+	?	?	+	Moderate	
Boscolo 2024	+	?	+	+	+	×	+	+	?	High	
**Carelli 2023**	+	+	×	+	+	+	?	+	+	Low	Primary
Cavayas 2018	+	+	+	+	+	×	×	+	?	High	
Gao 2023	+	?	×	+	+	×	?	+	+	High	
Garcia 2013	?	?	×	×	?	×	×	×	?	High	
Grasselli 2017	?	+	×	+	+	?	?	×	+	High	
Haneke 2016	×	+	×	+	+	?	?	+	+	Moderate	
Hsu 2009	+	+	×	+	+	?	?	+	+	Moderate	
Joshi 2024	+	?	×	+	+	+	?	×	+	Moderate	
Juthani 2018	+	+	×	+	+	?	?	?	+	Moderate	
Kang 2025	+	?	×	+	+	×	?	+	+	High	
Kim 2016	+	×	×	+	+	?	?	+	×	High	
Kim 2017	+	+	×	+	+	?	?	+	+	Moderate	
Kızmaz 2023	+	+	×	+	+	?	?	+	+	Moderate	
Ko 2020	+	+	×	+	+	?	?	+	+	Moderate	
Lee 2022	+	+	+	+	+	?	?	+	+	Low	
Li 2023	+	+	+	+	+	×	?	?	+	High	
Menaker 2019	+	+	+	+	+	?	?	+	+	Low	
**Na 2018**	+	+	×	+	+	+	?	+	+	Low	Primary
**Nesseler 2024**	+	+	+	+	+	?	?	+	?	Moderate	Primary
Ortuño 2025	×	?	×	+	+	?	?	×	?	High	
**Pieri 2012**	+	?	×	+	+	×	+	+	+	High	Primary
**Pieri 2013**	+	+	×	+	+	?	+	+	+	Low	Sens.
Pluim 2012	+	+	+	+	+	×	×	+	?	High	
Poth 2022	+	+	+	+	+	×	?	+	+	Moderate	
Poth 2024	+	+	+	+	+	?	?	+	+	Low	
Quintana 2021	+	+	+	+	+	?	?	+	+	Low	
**Schmidt 2012**	+	+	×	+	+	+	?	+	+	Low	Primary
Schweickhardt 2023	+	+	×	+	×	×	?	×	+	High	
Selçuk 2021	+	+	×	?	?	?	?	×	+	High	
Silvetti 2019	+	+	×	+	+	?	?	+	+	Moderate	
**Sun 2010**	+	+	+	+	+	?	?	+	+	Low	Sens.
Suzuki 2023	×	+	+	?	?	×	×	?	?	High	
Sysiak-Sławecka 2023	+	+	×	+	+	×	?	+	+	High	
Thomas 2017	+	+	×	+	+	+	+	?	+	Low	
**Uçar 2024**	+	+	×	+	+	+	?	+	+	Low	Primary
Wang 2020	+	+	×	+	+	?	?	+	+	Moderate	
Wang 2021	+	+	+	+	+	?	?	+	+	Low	
Wang 2023	+	+	×	+	+	?	?	×	+	Moderate	
Wells 2025	+	+	×	×	×	×	?	?	+	High	
Xu 2022	+	+	×	+	+	+	?	×	+	Moderate	
**Yang 2022**	+	+	×	+	+	+	?	+	+	Low	Primary
Yun 2021	+	+	+	+	×	?	?	+	+	Moderate	
Zhao 2025	+	+	×	+	×	×	?	+	?	High	

Q1 sample frame; Q2 sampling; Q3 sample size ≥246; Q4 subjects and setting described; Q5 coverage of the identified sample;
Q6 valid identification of the condition; Q7 standard, reliable measurement; Q8 appropriate statistical analysis; Q9 response rate / exclusions.
Concern: low ≥7 items met; moderate 5–6; high ≤4; one category added when Q6 is not met. Bold, Outcome B study (Tier A, k = 7);
Sens., Outcome B sensitivity analysis only.

+ Yes ? Unclear × No

Q1 sample frame; Q2 sampling; Q3 sample size ≥246; Q4 subjects and setting described; Q5 coverage of the identified sample; Q6 valid identification of the condition; Q7 standard, reliable measurement; Q8 appropriate statistical analysis; Q9 response rate/exclusions. Overall concern: low, ≥7 items met; moderate, 5–6; high, ≤4; one category added when Q6 is not met. Bold, Outcome B study (Tier A, k = 7); Sens., Outcome B sensitivity analysis only. JBI, Joanna Briggs Institute.
